# Neurotrophic Activity and Its Modulation by Zinc Ion of a Dimeric Peptide Mimicking the Brain-Derived Neurotrophic Factor N-Terminal Region

**DOI:** 10.1021/acschemneuro.2c00463

**Published:** 2022-11-08

**Authors:** Lara Russo, Chiara Giacomelli, Mariagrazia Fortino, Tiziano Marzo, Gianmarco Ferri, Mariantonietta Calvello, Alessandro Viegi, Antonio Magrì, Alessandro Pratesi, Adriana Pietropaolo, Francesco Cardarelli, Claudia Martini, Enrico Rizzarelli, Laura Marchetti, Diego La Mendola, Maria Letizia Trincavelli

**Affiliations:** † Dipartimento di Farmacia, Università di Pisa, Pisa 56127, Italy; ‡ Università di Catanzaro, Catanzaro 88100, Italy; § Laboratorio NEST, Scuola Normale Superiore, Pisa 56127, Italy; ∥ Bio@SNS, Scuola Normale Superiore, Pisa 56126, Italy; ⊥ Istituto di Cristallografia, Consiglio Nazionale delle Ricerche (CNR), Catania 95126, Italy; # Dipartimento di Chimica e Chimica Industriale, Università di Pisa, Pisa 56124, Italy; ∇ Università degli Studi di Catania, Catania 95124, Italy

**Keywords:** BDNF, peptide mimetics, TrkB, dimer, zinc, neurite outgrowth

## Abstract

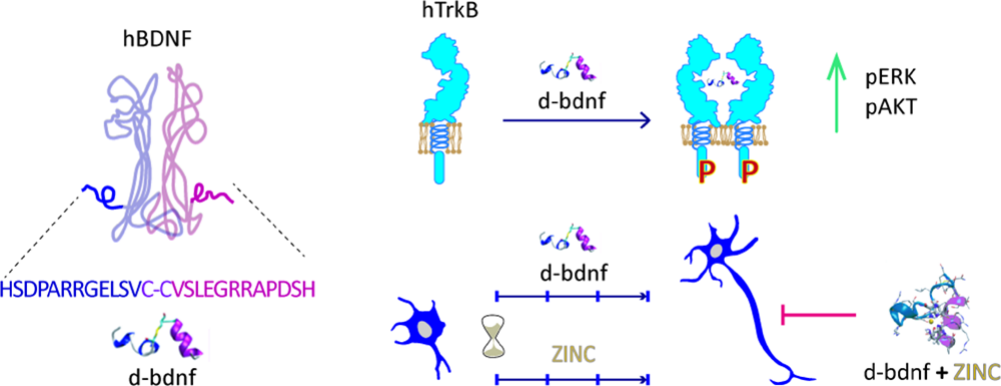

Brain-derived neurotrophic factor (BDNF) is a neurotrophin (NT) essential for neuronal development and synaptic plasticity. Dysregulation of BDNF signaling is implicated in different neurological disorders. The direct NT administration as therapeutics has revealed to be challenging. This has prompted the design of peptides mimicking different regions of the BDNF structure. Although loops 2 and 4 have been thoroughly investigated, less is known regarding the BDNF N-terminal region, which is involved in the selective recognition of the TrkB receptor. Herein, a dimeric form of the linear peptide encompassing the 1–12 residues of the BDNF N-terminal (d-bdnf) was synthesized. It demonstrated to act as an agonist promoting specific phosphorylation of TrkB and downstream ERK and AKT effectors. The ability to promote TrkB dimerization was investigated by advanced fluorescence microscopy and molecular dynamics (MD) simulations, finding activation modes shared with BDNF. Furthermore, d-bdnf was able to sustain neurite outgrowth and increase the expression of differentiation (NEFM, LAMC1) and polarization markers (MAP2, MAPT) demonstrating its neurotrophic activity. As TrkB activity is affected by zinc ions in the synaptic cleft, we first verified the ability of d-bdnf to coordinate zinc and then the effect of such complexation on its activity. The d-bdnf neurotrophic activity was reduced by zinc complexation, demonstrating the role of the latter in tuning the activity of the new peptido-mimetic. Taken together our data uncover the neurotrophic properties of a novel BDNF mimetic peptide and pave the way for future studies to understand the pharmacological basis of d-bdnf action and develop novel BDNF-based therapeutic strategies.

## Introduction

The neurotrophin (NT) family of growth factors consists of nerve growth factor (NGF), brain-derived neurotrophic factor (BDNF), neurotrophin 3 (NT-3), and neurotrophin 4 (NT-4).^1^ NTs are critical mediators of neuron survival and development in the peripheral and central nervous systems (CNS). They exert their function generally acting as homodimers held together by non-covalent bonds and binding two different membrane receptors: the p75 NT receptor (p75^NTR^) common to all NTs, and the tropomyosin receptor kinase (Trk) family that selectively recognizes different NTs (TrkA for NGF, TrkB for BDNF and NT-4, and TrkC for NT-3).^2^

BDNF is the predominant NT in the adult brain and is widely distributed in the cortical areas, visual cortex, and hippocampus.^3^ Activation of TrkB by BDNF initiates downstream signaling cascades mediated by Ras/ERK, PI3K/Akt, and PLC-γ.^4^ After its activation, the BDNF–TrkB complex undergoes endocytosis, recycling, or degradation and crucially takes the axonal transport route to sustain neuron survival and differentiation.^5^ Furthermore, BDNF/TrkB signaling regulates dendritic branching, the density of spines, and spine morphological specializations, acting both as the mediator and modulator of synaptic plasticity and communication in the CNS.^6,7^ Intriguingly, several different molecules such as ATP, G-protein-coupled receptor ligands and metal ions can modulate either NTs or their receptors, thus enriching the panorama of signaling cues involved in neurotrophic support.^8,9^ For the BDNF–TrkB signaling axis, an interesting role is played by zinc, an essential metal ion in brain physiology.^10^ Zinc is released from synaptic vesicles of glutamatergic neurons reaching concentrations up to 100 μM in the synaptic cleft,^11,12^ where it was reported to prompt post-synaptic, BDNF-independent TrkB activation.^13^ Zinc transactivates TrkB promoting its phosphorylation through the activation of the Src family kinase by relieving their TrkB inhibition. Less is known regarding the direct effect of zinc on mature BDNF, it was only recently reported that zinc binds the pro-domain of the unprocessed BDNF precursor, in particular its Val66Met polymorphic variant^14^ modulating synaptic remodeling and plasticity.

The modulation of the BDNF–TrkB signaling axis has been recently postulated as a drug target for the treatment of neurological diseases, including nerve injury, neurodegenerative diseases, and neuropsychiatric disorders.^15,16^ Unfortunately, the therapeutic use of BDNF, as that of other NTs, is challenging due to the uncontrolled side effects as well as their low bioavailability.^17^ To overcome such limitations, the use of peptide fragments that can mimic specific domains of the NTs has been explored in the last few years.^18^ The most investigated BDNF structural elements have been so far loop 2^19,20^ and loop 4^21^ to achieve TrkB activation and the PAKKR sequence that recognizes the p75NTR receptor.^22^ Interestingly, dimeric versions of these peptides (e.g., the dimeric BM17d99^23^ or GSB-106^24^ peptides) were reported to display an improved ability to activate TrkB and promote neuronal survival.

Previous functional studies agree in identifying the N-terminal region as a pivotal NT domain for the binding selectivity and activation of Trks.^25−27^ However, most of these information were obtained from the NGF-TrkA system.^28,29^ On the other hand, the BDNF N-terminal region has been less investigated. Our group previously reported that a linear peptide fragment encompassing the 1–12 residues of BDNF N-terminal displays proliferative capacity in undifferentiated cells; however, the relevance of this observation in the context of neurodevelopment has not been assessed, yet.^30,31^ Moreover, whether zinc also modulates the neurotrophic activity of the BDNF peptide-mimetics, as documented for the full-length proBDNF protein, has remained so far mostly unexplored.

Herein, peptides that mimic the BDNF N-terminal region were investigated. We demonstrate that a dimeric peptide (d-bdnf) bearing two BDNF(1–12) pairs was able to activate TrkB promoting a neurotrophic effect. Interestingly, zinc coordination by d-bdnf inhibited the peptide effects, thus suggesting that the metal ion may tune the activity of d-bdnf at synapses or brain regions where zinc concentration is high.

## Results and Discussion

### Biochemical and Molecular Basis of TrkB Receptor Activation by the d-bdnf Peptide

**Figure 1:**
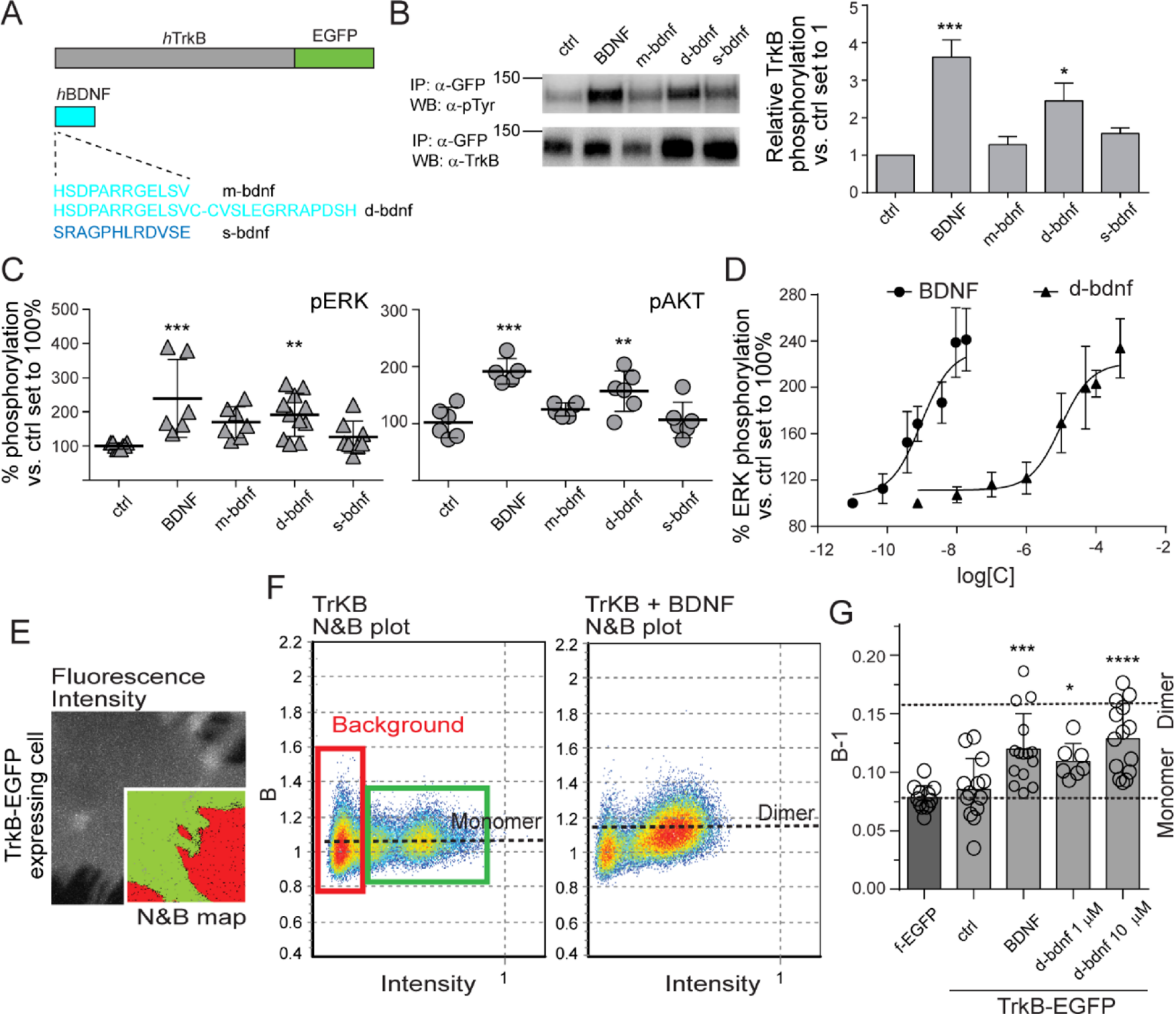
Activation of the TrkB receptor by BDNF and its mimetic peptide ligands. (A) Schematic picture of the human TrkB receptor fused to the EGFP construct (hTrkB-EGFP); recombinant human mature BDNF (hBDNF); monomeric peptide, m-bdnf; dimeric peptide, d-bdnf; scrambled s-bdnf. (B) WB showing Tyr phosphorylation (p-Tyr, top) and total TrkB levels (bottom) in SH-SY5Y cells transfected with hTrkB-EGFP in the absence (ctrl) or presence of 10 min stimulation with 150 ng/mL BDNF or 10 μM m-bdnf, d-bdnf, or s-bdnf. Samples were immunoprecipitated (IP) with the anti-GFP antibody before WB. The relative density of the bands is reported on the right of the blots, as mean ± SEM of five (BDNF) and four (peptides) independent replicas; each p-Tyr band density was divided for the respective TrkB band and normalized to the ctrl signal (***p < 0.001, *p < 0.05 vs ctrl, according to one-way ANOVA with Bonferroni’s multiple comparison test). (C) Scatter dot plot of ERK (left) and AKT (right) phosphorylation levels detected in differentiated SH-SY5Y cells, after stimulation with the same treatments as in (B). Phosphorylation levels were normalized to the cell number and reported as percentage versus the ctrl. Data ± SD were pooled from three independent replicas performed in duplicate. (D) ERK phosphorylation dose–response curve of BDNF and d-bdnf. Data ± SEM were pooled from two independent replicas performed in duplicate. (E) Intensity image of a TrkB-EGFP transfected cell (scale bar 10 μm). White square defines a region of the image that contains both the cell membrane and the background signal. N&B was calculated for the white square and results were displayed according to the color code in panel E. (F) Example of N&B results obtained for a TrkB-EGFP-transfected cell before (left) and after (right) stimulation with BDNF. The dashed black lines identify the mean values of B for the monomer and the dimer, as indicated. (G) Bar graph of mean ± SD ‘B-1’ values retrieved from N&B analysis for all the different conditions measured. The horizontal dashed lines indicate the ‘B-1’ average values assigned to the monomer (based on data from f-EGFP) and the dimer. Data (round circles) were pooled from two independent experiments. A one-way ANOVA with Dunnett’s multiple comparisons test was applied to compare data to the f-EGFP reference (*p < 0.05, ***p < 0.001, ****p < 0.0001).

The possibility of mimicking the function of the full-length BDNF protein by using a linear peptide fragment encompassing the first 12 amino acids of the mature NT N-terminal was already postulated.^31^ Here, we named this sequence as m-bdnf (Figure 1A) and performed a systematic comparison between the performance of the full-length human BDNF produced recombinantly in *Escherichia coli*(32) and (i) m-bdnf, (ii) a dimeric peptide displaying two copies of the m-bdnf sequence covalently bound by a disulfide bridge (d-bdnf), and (iii) a monomeric peptide displaying the scrambled sequence of m-bdnf (s-bdnf). We first investigated the ability of these peptides to activate the TrkB receptor. To this purpose, we expressed a recombinant GFP fusion of human TrkB (Figure 1A) in SH-SY5Y cells. Then, cells were stimulated with BDNF or the related peptides. We pulled down the receptor from cell lysates by immunoprecipitation and probed its phosphorylation by the western blot, showing that only d-bdnf was able to promote a significant TrkB phosphorylation (*p* < 0.05) when administered at μM concentrations, although not at comparable levels of the nM BDNF concentration (Figure 1B). Nevertheless, this was sufficient to prompt robust phosphorylation of downstream effectors ERK and AKT; indeed, as shown in Figure 1C, only BDNF and d-bdnf were able to induce a significant ERK and AKT phosphorylation increase with respect to the unstimulated control, in cell-based assays similar to those previously used to test the activity of TrkB agonists.^33^ Then, the same cell-based assays for ERK phosphorylation were used to better elucidate the agonist properties of d-bdnf (Figure 1D). As expected, BDNF showed an EC_50_ value of 0.69 ± 0.24 nM (referred to the monomer form) in accordance with the literature data.^15^ The d-bdnf peptides showed an EC_50_ value of 13.63 ± 3.87 μM. Interestingly, the BDNF and d-bdnf maximal effects (*E*_max_) of ERK phosphorylation did not significantly differ (237.1 ± 5.0; 227.3 ± 6.3 for BDNF and d-bdnf, respectively). The same result was not obtained when BDNF and d-bdnf were administered to HEK 293 cells, which do not express TrkB (Figure S1A). Taken together, these results highlight that the dimeric peptide acts as a specific agonist of TrkB, although at higher concentrations with respect to the full-length NT. Then, to better elucidate if d-bdnf could act as a partial antagonist or produce additive effects, competition experiments were done (Figure S1B). Specifically, an EC_50_ concentration of BDNF (1 nM) or of d-bdnf (10 μM) was used in combination with the increasing concentration of d-bdnf and BDNF, respectively, and the increase of ERK phosphorylation was assessed. As reported, both combinations were able to reach the maximal effects similar to that of BDNF alone with no significant differences. These data highlight that d-bdnf had neither antagonist nor additive effects. Of note, we also performed a non-permeabilizing immunofluorescence analysis to quantify the membrane-bound BDNF in differentiated SH-SY5Y cells in the absence or presence of BDNF and d-bdnf (Figure S1C,D). A 1 h treatment with a saturating dose of BDNF (150 ng/mL) led to a ∼40% increase of the fluorescent signal on the cells, demonstrating that endogenous BDNF produced by the cell line does not saturate TrkB in our experimental setup. The same increment was not observed upon treatment with the d-bdnf peptide, while instead we measured a slight (∼18%) decrease of the signal possibly due to the ability of the peptide to displace endogenous BDNF at the tested dose (10 μM). Interestingly, d-bdnf co-administered with BDNF was able to decrease by ∼16% membrane-bound BDNF levels, further suggesting the possibility that the two compete for the same binding sites on the TrkB receptor. Future analysis, however, will be necessary to understand if d-bdnf occupies the same orthosteric site of BDNF.

The same TrkB-GFP expressing SH-SY5Y cells were used to deeper investigate the mechanism of TrkB activation induced by d-bdnf, using an advanced imaging approach in living cells. We exploited a fluorescence correlation technique, which directly correlates the brightness of each fluorescent pixel of the image to the number of TrkB molecules averagely populating that pixel during the observation time (number & brightness technique—N&B^34,35^). By analyzing the N&B map for different regions of the cell corresponding to the TrkB membrane pool (inset of Figure 1E), we derived the fraction of pixels corresponding to monomeric, dimeric, and oligomeric receptors (Figure 1F) either in unstimulated conditions or after treatment with BDNF or d-bdnf. A monomeric GFP construct used as a reference allowed us to conclude that while TrkB-EGFP is present almost exclusively in the monomeric form at the plasma membrane in resting conditions, there is a shift toward the dimeric form similarly induced by the addition of BDNF and d-bdnf, especially when the latter is administered at the highest concentration (Figure 1G). Overall, these data suggest that the d-bdnf peptide can support, at the investigated concentrations, an appreciable TrkB surface activation; however, similar to what was observed for the BDNF stimulation, this does not seem to imply a stable receptor oligomerization. Rather, activation is likely to occur in a dynamic equilibrium between monomers and dimers, so that a complete dimer population cannot be obtained for any of the ligands/concentrations investigated. In this scenario, it is, therefore, possible that both BDNF and d-bdnf bind and activate both the monomer and dimeric forms of TrkB, in agreement with previously reported data.^36^

**Figure 2:**
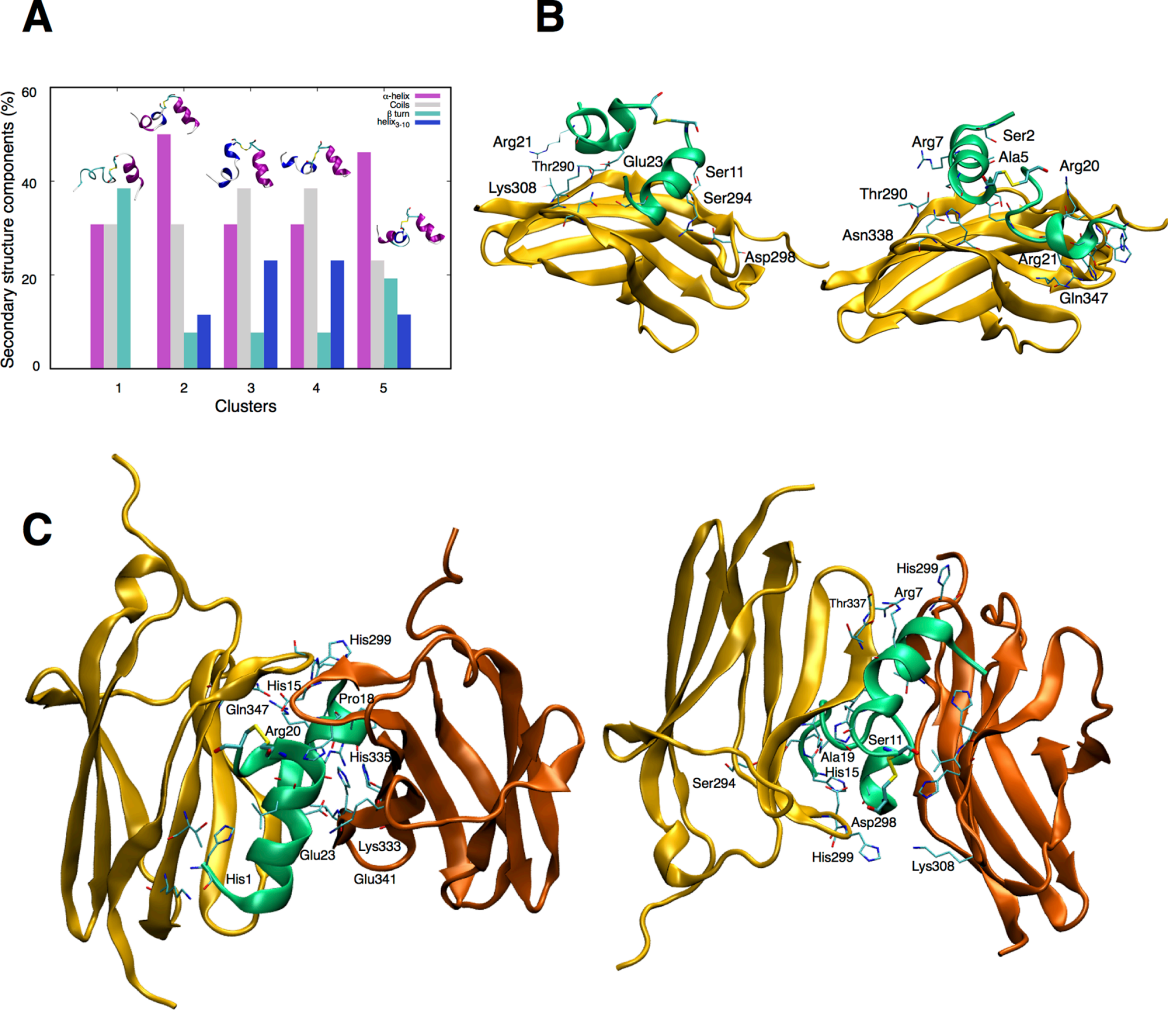
Molecular dynamics of the d-bdnf structure alone or with TrkB. (A) Five most representative clusters along with a histogram representing the percentage of secondary structure components. N- and C-terminal residues are oriented from the left to the right side. (B) Snapshots of the two most representative TrkB-D5/d-bdnf binding poses. TrkB is reported in gold whereas d-bdnf is reported in green. Residue labels highlight the amino acids involved in the non-covalent interactions. (C) Snapshots of the two main TrkB1-d-bdnf-TrkB2 binding poses. TrkB1 is reported in gold, TrkB2 is reported in orange whereas d-bdnf is reported in green. Residue labels highlight the amino acid residues involved in the non-covalent interactions.

To deeper investigate the possible modes of TrkB extracellular domain (ECD) recognition by d-bdnf, MD simulations were performed. The conformational features of d-bdnf were obtained from parallel tempering simulations, and the most representative five clusters are reported in Figure 2A, along with a histogram representing the percentage of secondary structure components.

Notably, the dimeric peptide scaffold of d-bdnf preserves the alpha-helix content differently from the m-bdnf previously investigated.^31^

The predicted binding poses of the TrkB receptor with d-bdnf are reported in Figure 2B, in which the compact binding pose on the left side was predicted to be more stable than the pose with the peptide oriented in an extended conformation on the right, by 4 kcal/mol. Several non-covalent interactions largely involving hydrogen bonds have been found. Table S1 summarizes the main hydrogen bonds observed in the analyzed conformations, indicating the amino acid residues participating in this weak interaction. Asp298, Thr290, and Asn338 are the residues belonging to the TrkB receptor largely involved in non-covalent interactions with Ser2, Arg7, Ser11, and Glu23 of d-bdnf.

We then proceeded to assess the binding poses of the d-bdnf in complex with two chains of TrkB, starting from the binding poses sketched in Figure 2B. Results are shown in Figure 2C, and the network of hydrogen bonds is reported in Table S2. In line with the binding with one receptor chain, one TrkB receptor contacts the d-bdnf peptide via Ser294, Asp298, His299, Lys308, Asn338, and Gln347. The second chain of TrkB faces the d-bdnf and maintains a lower similarity with the receptor monomer binding modes, specifically through Asp298, Lys308, His343, and His299. The dimer peptide contacts the receptor mainly via Ser2, Arg7, Ser11, Arg20, Arg21, and Ser25.

The reported data suggest an asymmetric interaction of the d-bdnf with the TrkB receptor, since one chain of TrkB is more prone to contact the peptide. On the whole, d-bdnf is predicted to bind with one chain of TrkB, leaving the second half to weakly interact with a second chain of the TrkB receptor. These data are in agreement with the transient dimerization of TrkB in the presence of d-bdnf measured in living cells by the N&B technique (Figure 1G).

### Evaluation of BDNF Peptido-Mimetic Neurotrophic Activity

**Figure 3:**
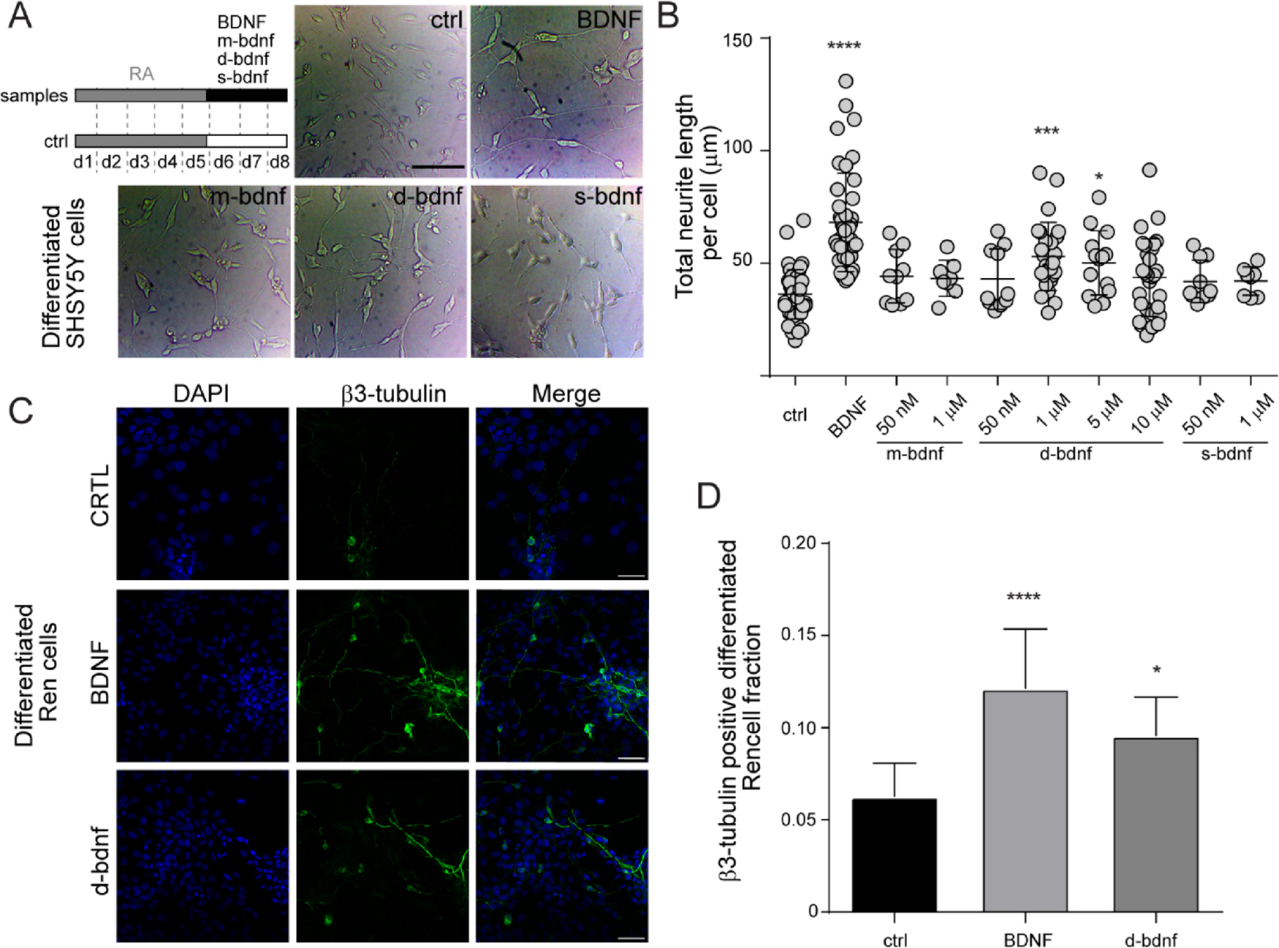
Neurite outgrowth stimulated by BDNF and its mimetic peptide ligands. (A) Top, left: schematic timeline of the experiment. SH-SY5Y cells were differentiated for 5 days (d1–5) by addition of retinoic acid (RA, gray bar) in the cell medium. Then, cells were stimulated for 3 days (d6–8) with 150 ng/mL BDNF or different concentrations of its peptide-mimetics (black bar) or left untreated (white bar). On the right and bottom, representative images of the differentiated cells at the end of each treatment. Scale bar 50 μm. (B) Scatter dot plot of the total neurite length, normalized to the number of cell bodies present in each analyzed field. Each sample comprises fields (*n*_fields_) pooled from three or four independent replicas (*n*_fields_ for CTRL: 43; BDNF: 42; m-bdnf 50 nM: 10; m-bdnf 1 μM: 8; d-bdnf 50 nM: 10; d-bdnf 1 μM: 26; d-bdnf 5 μM: 13; d-bdnf 10 μM: 29; s-bdnf 50 nM: 10; s-bdnf 1 μM: 8). For each field, an average of 50–200 cells were counted. The horizontal line represents the average value, and the bars are the SD (****p < 0.0001, ***p < 0.001, *p < 0.05 according to the one-way ANOVA with Dunnett’s multiple comparison test). (C) Representative fluorescence images of DIV3 Ren cells stained with DAPI (blue) and β3-tubulin (green) after differentiation with the standard medium (CTRL), or with the standard medium supplemented with 50 ng/mL BDNF or with 1 μM d-bdnf. Scale bar: 50 μm. (D) Column plot of the mean ± SD fraction of β3-tubulin (green) positive on the total cells, counted in the DAPI channel of the analyzed fields (*n*_fields_) (CTRL: *n*_fields_ = 10 and total *n*_cells_ = 4756; BDNF: *n*_fields_ = 11 and total *n*_cells_ = 4330; d-bdnf: *n*_fields_ = 10 and total *n*_cells_ = 5619 cells; ****p < 0.0001, *p < 0.05 according to the one-way ANOVA with Dunnett’s multiple comparison test).

We next tested the efficiency of neurotrophic activity displayed by BDNF peptides, in comparison to the full-length BDNF. To this purpose, we used differentiated SH-SY5Y cells as a bona fide model of neuronal differentiation.^37^ We used a two-step differentiation protocol (Figure 3A, top left), in which the cells were first treated with retinoic acid for 5 days, and then incubated for 3 days with BDNF or different concentrations of m-, d-, and s-bdnf peptides. Cells were observed at the optical microscope, and the emitted neurite network in the various samples was quantitatively evaluated.^32^ Obtained data showed that the stimulation with d-bdnf, but not with m- or s-bdnf, at 1 and 5 μM concentrations is able to elicit a neurite network similar to that raised by nM concentrations of BDNF (Figure 3B). These data suggest that the TrkB binding and activation promoted by d-bdnf (Figures 1 and 2) is effective in producing neurotrophic activity. Higher doses of the same peptide were not able to produce the same effect; however, this cannot be explained by the toxic effects of the peptide at these concentrations. Indeed, a viability assay of cells treated in the same conditions demonstrated no reduced vitality of the culture in the presence of 5–10 μM d-bdnf; rather, we detected some toxicity exerted by lower doses of m-bdnf, which may help to explain its reduced performance with respect to d-bdnf (Figure S2). We can thus conclude that higher concentrations of d-bdnf (e.g., 10 μM) do activate pTrkB and other pathways in relatively short-term assays (Figures 1 and S1) but are less effective in relatively longer-term assays such as neurite outgrowth. This could likely be related to the kinetics or duration of TrkB activation prompted by d-bdnf, or to biased signaling, or desensitization, and could be matter of future investigations.

Interestingly, in a recent report, the ability of BDNF to positively modulate the differentiation of a human neural progenitor cell line (RenVM), derived from the ventral mesencephalic region of the developing human brain, into functional neurons was reported.^38,39^ Thus, we implemented the assessment of d-bdnf neurotrophic activity by evaluating its ability in increasing the neuronal cell fraction of differentiated Ren cells with respect to BDNF (Figure 3C,D). The results corroborated the d-bdnf neurotrophic activity as evidenced by the significant increase of detected neurons with respect to untreated cells, in a similar way to BDNF.

**Figure 4:**
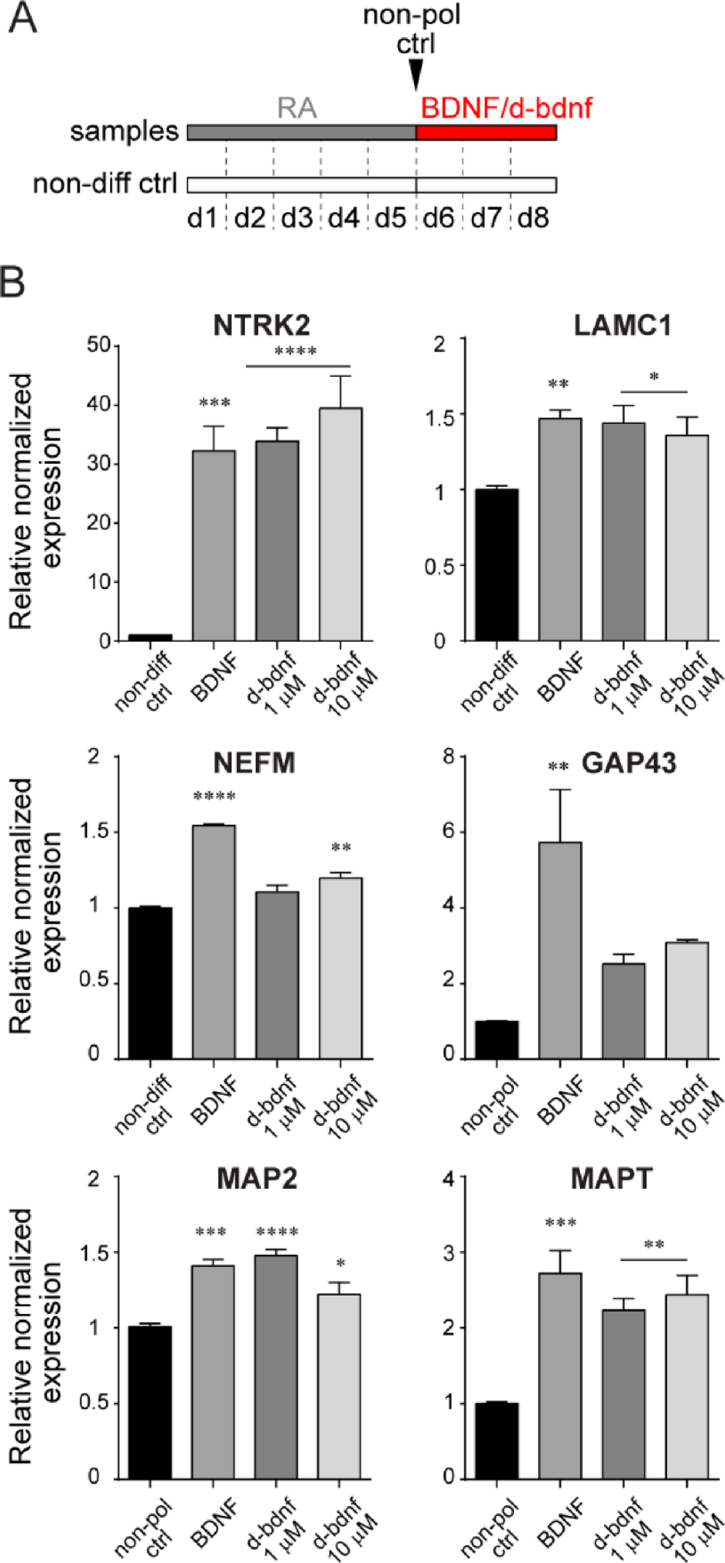
Neuronal differentiation and polarization genes stimulated by BDNF and d-bdnf mimetic peptide. (A) Schematic timeline of the experiment. As non-differentiated control, we used untreated cells seeded at the same density (non-diff ctrl, white bars). As a control for the expression of neuronal polarization genes, we used cells stopped at d5 (non-pol ctrl). (B) RT-PCR analysis of the mRNA expression levels of neuronal differentiation markers (NTRK2, LAMC1, and NEFM) and polarization markers (GAP43, MAP2, and MAPT) induced by BDNF and d-bdnf with respect to non-diff and non-pol ctrl samples, respectively. Data are expressed as fold changes with respect to the average ctrl value set to 1, and the mean ± SEM of four replicas from two independent experiments are reported (****p < 0.0001, ***p < 0.001, **p < 0.01, and *p < 0.05 according to the one-way ANOVA with Bonferroni’s multiple comparison test).

To understand if the BDNF and d-bdnf share similar mechanisms of differentiation, we next performed a gene expression analysis of differentiated SH-SY5Y cells (Figure 4A). As shown in Figure 4B, we found that d-bdnf was able to promote, to a similar extent of BDNF, the expression of NTRK2 and LAMC1 genes, two genes typically upregulated in differentiated versus non-differentiated SH-SY5Y.^40,41^ Also, when analyzing genes involved in neuronal polarization, which is a feature specifically acquired by SH-SY5Y cells in the last 3 days of the NT treatment,^37^ we found that d-bdnf could prompt the expression of genes involved in dendrite (MAP2) and axon (MAPT) specification to a similar extent to BDNF. Nevertheless, NEFM gene expression was significantly increased only at the highest d-bdnf dose (10 μM); and GAP43, a recognized marker of developing and regenerating axons, was not significantly increased by either the d-bdnf doses tested. The latter is probably the most evident difference in the gene expression pattern induced by BDNF and d-bdnf. A possible explanation for this difference is the lack of p75^NTR^ activation prompted by d-bdnf stimulation when compared to BDNF. Indeed, p75^NTR^ has a fundamental role in axon outgrowth and branching.^42,43^

### Zinc Modulation of Peptido-Mimetic Neurotrophic Activity

**Figure 5:**
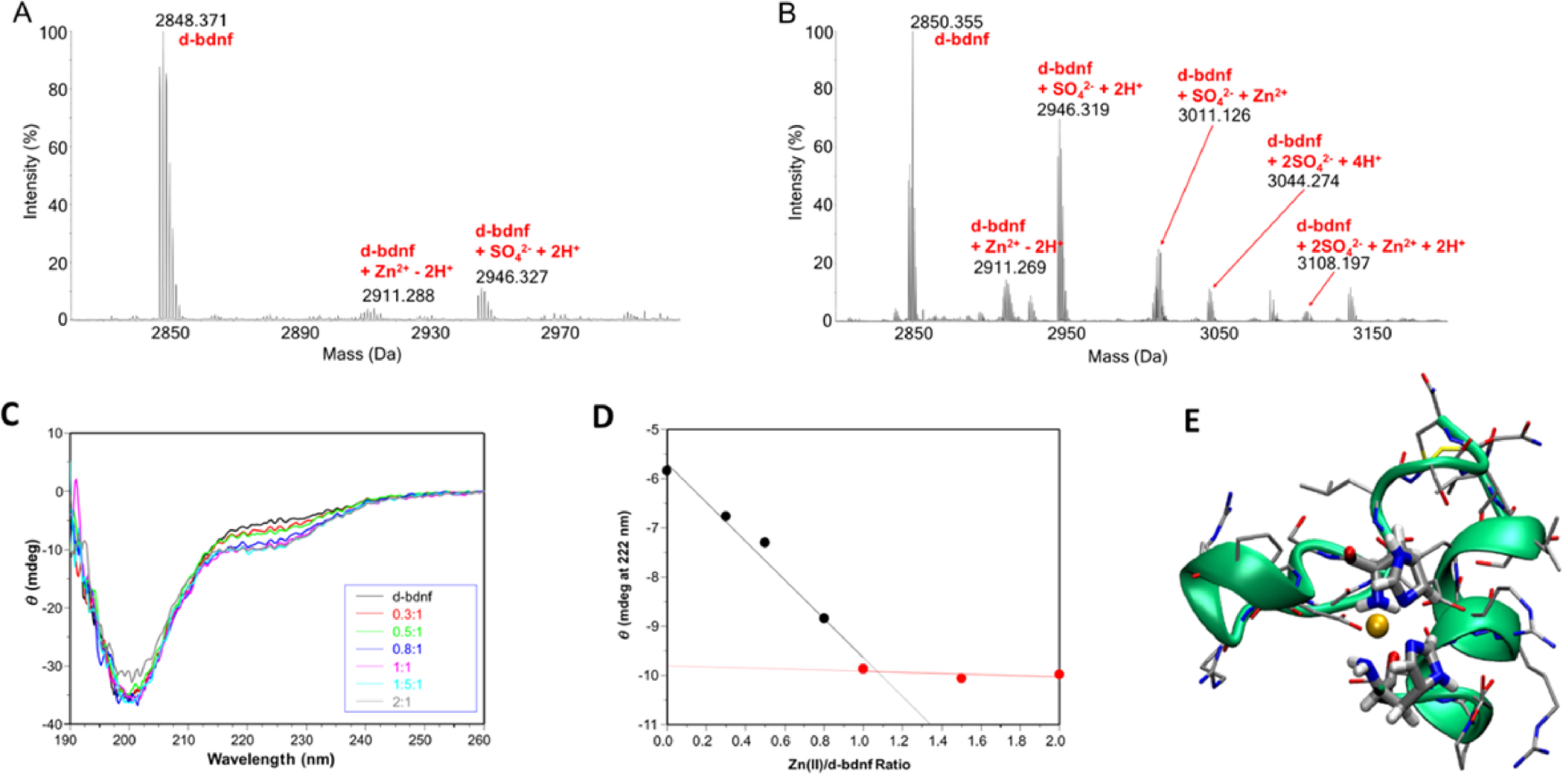
Evaluation of Zn^2+^ ion binding on d-bdnf. (A,B) Deconvoluted ESI mass spectra of 10^–6^ M d-bdnf solutions incubated in water 2 h, 37 °C in the presence of a 100-fold excess of Zn^2+^. 0.1% of formic acid was added (A) or not (B) just before the infusion. (C) Far-UV CD spectra at pH 7.4 for titration with Zn^2+^ of d-bdnf (the black curve is the spectrum of peptide; red, green, blue, cyano, and gray are spectra of peptide after the addition of increasing equivalents of metal ions. (D) Spectral changes as a function of zinc addition monitored at λ = 222 nm. [d-bdnf] = 1 × 10^–5^ M. (E) Most representative conformation for the BDNF(1–12) dimer coordinating Zn(II) metal ion. Carbon atoms are shown in silver, nitrogen atoms are reported in blue, oxygen atoms in red, sulfur atoms are represented in yellow, and Zn atom is reported in orange. The secondary structure of the peptide is represented in green.

Considering the capability of zinc ion to modulate both TrkB and proBDNF synaptic activity,^13,14^ we investigated whether and how Zn^2+^ ions could modulate the neurotrophic activity of d-bdnf peptide. Indeed, metal binding to the m-bdnf was previously demonstrated.^30^ To this purpose, we first performed high-resolution mass spectrometry on d-bdnf (Figure 5A,B) and m-bdnf (Figure S3) to gain precise information on the nature of the adducts that are formed, including the binding stoichiometry.^44,45^Figure 5A reports the spectrum obtained for d-bdnf after 2 h of incubation with the excess of Zn^2+^ and addition of 0.1% formic acid immediately before injection to enhance the ionization of the investigated molecules. We can observe a modest formation of adducts featured by one bound Zn^2+^ ion. Specifically, the peak at 2911.288 Da (Figure 5A) is assignable to the d-bdnf that coordinates one zinc atom. Assignments were validated through comparison of the measured and theoretical isotopic distributions (see Figure S4 for theoretical isotopic pattern and mass error calculation). In this respect, though the dimeric structure, the behavior of d-bdnf (Figures S3 and S4) was different from the expected coordination of two Zn^2+^ ions. Also, both for the m- and d-bdnf cases, the relative percentages of the formed adducts is quite low and the main peak is that of the non-coordinated peptides. Noteworthy, a signal at 2946.327 Da corresponding to the adduct with the sulphate ion, deriving from incubation with ZnSO_4_, is present in case of d-bdnf (Figure 5A). In the second experimental set, we performed the same analysis without the addition of formic acid before injection (Figure 5B). In fact, a more acidic environment could perturb the coordination equilibria due to the protonation of those amino acid residues, that is, histidine.^45,46^ It appears clear that the addition of the acid significantly affects the amount of the formed adducts, as the solution analyzed without formic acid displays a greater amount of mono-metalated peptides. This observation allows to surmise that the binding of the metal likely occurs at the level of the terminal His residues. The formic acid increased His protonation limiting the amount of the peptide-zinc adduct. Conversely, without the addition of acid, His remained available for the coordination toward the metal center resulting in a greater amount of adducts. This interpretation is in nice agreement with recent reports by Wang et al.^14^ Accordingly, a plethora of secondary mixed adducts with Zn^2+^ and the sulphate ions was also detectable (Figures 5B and S3, bottom). Interestingly, despite the presence of multiple adducts with sulphate ions, no multiple adducts with Zn^2+^ ions were detected, further strengthening the hypothesis that the His residue can likely represent the main zinc binding site. The experimental evidence that both the m-bdnf peptide and d-bdnf coordinate one metal center—despite the presence of two available histidine residues—might be attributed to the higher conformational freedom compared with m-bdnf, leading to folded structures (Figure 2A) that impair the coordination of additional Zn^2+^ ions.

The far-UV CD spectra of peptide d-bdnf show a wide minimum around 200 nm a typical feature of predominantly a random coil conformation (Figure 5C). The minimum band at 222 nm is related to the contribution of a side-chain chromophore and in particular of imidazole.^47^ The addition of increasing amount of Zn^2+^ does not significantly change the dichroic band suggesting that the metal coordination environment does not involve backbone nitrogen atoms but only the side chain groups.

The addition of increasing amount of Zn^2+^ shows a decrease in the minimum of the band centered at λ = 222 nm and reaches a plateau after the addition of one molar equivalent of Zn^2+^ (Figure 5D). The observed changes are due to the binding of imidazole to zinc ion, and the trend is in agreement with a 1:1 metal to ligand stoichiometry as observed in the ESI MS spectra.

Potentiometric measurements were carried out to give insight into the coordination environment of Zn^2+^ bound to the d-bdnf peptide (Figure S5). The protonation and complex stability constant values are reported in Table S3. The ligand displays eight protonation constants, and the *pK* values are similar to those reported for the monomer peptide according to the maintenance of similar conformational features.^31^ The four protonation equilibria of the aspartic and glutamic residues partly overlap but, in agreement with the literature data, the aspartic β-carboxylic group is more acidic than glutamic γ-carboxylic one.^48^ The potentiometric titrations were carried out exploring 0.9:1 and 2.2:1 metal to ligand molar ratio. Only mononuclear complex species were observed then the peptide d-bdnf is able to bind one zinc ion, according to ESI-mass and CD data. The peptide forms two complex species, [ZnLH] and [ZnL]. The first one is the predominant at pH = 6, and the calculated stability constant value [log*K*(111) = logβ(111) – log*K*(011) = 5.71] indicates the involvement of three nitrogen atoms in the metal coordination environment. The [ZnL] species display a stability constant value, logβ = 7.15, higher than that of protonated species indicative of the involvement of a further nitrogen atom in the metal binding mode. In this complex species, Zn^2+^ is bound to four nitrogen atoms with the involvement of two amino and two imidazole (2NH_2_,2N_Im_ coordination mode).

Precisely this coordination mode explains why d-bdnf does not bind two metal ions; the formation of macrochelate, that involves the nitrogen atoms of the N-terminal domain of the two chains simultaneously, prevents each single chain from bonds an ion with the same coordination of the monomer. In this regard, MD simulations were performed and the most representative conformation of d-bdnf coordinating the Zn^2+^ metal ion was reported in Figure 5E. Those predictions indicate a distorted tetrahedral coordination geometry of the Zn^2+^ metal ion within the d-bdnf. The Zinc coordination polyhedron involves two nitrogen atoms belonging to the two HIS1 residues of the d-bdnf and their NH_2_-groups. The oxygen atoms of the side chain belonging to ASP_3_ may assist this coordination. The root mean squared deviation from the apo-peptide is calculated as 4.9 Å, indicating a reorganization of the dimer upon the zinc ion coordination that involves mainly the N-terminal domain of the two chains, that remain flexible and in a disordered conformation, in agreement with the CD data.

**Figure 6:**
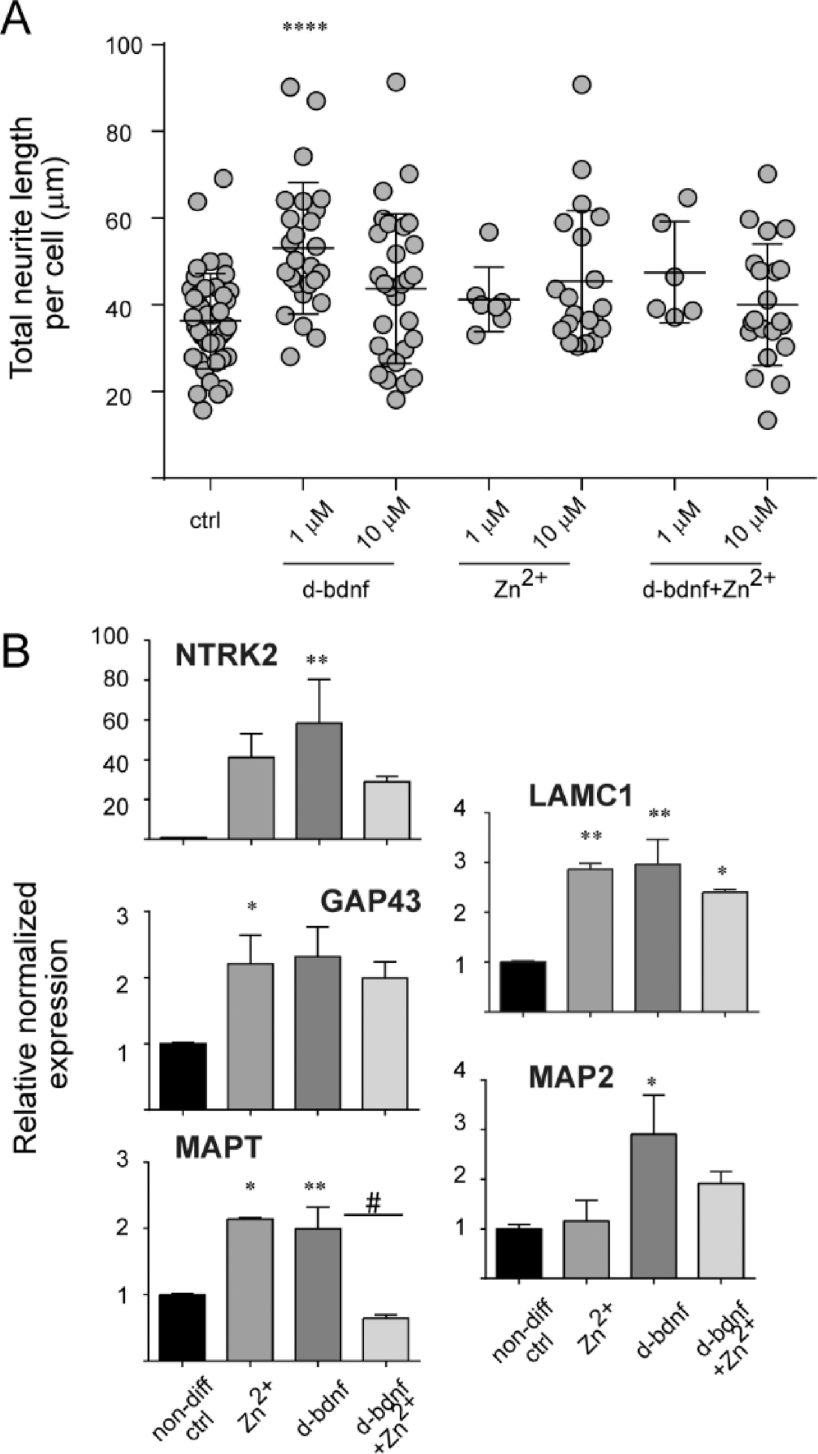
Modulation of d-bdnf neurotrophic activity by Zn^2+^ ion. (A) Neurite outgrowth assay for d-bdnf-Zn^2+^ conjugate. The ctrl and d-bdnf samples are the same as in Figure 2C, here reported as a reference. Data represent the mean ± SD of fields pooled from two independent replicas. (****p < 0.0001, according to the one-way ANOVA with Dunnett’s multiple comparison test). (B) RT-PCR analysis of the mRNA expression levels of the marker genes (as described in Figure 3B). Data are expressed as fold changes with respect to the average ctrl value set to 1, and the mean ± SEM of four replicas from two independent experiments are reported (**p < 0.01, *p < 0.05, vs ctrl; #*p* < 0.05 vs d-bdnf, according to one-way ANOVA with Bonferroni’s multiple comparison test).

The obtained metalation data prompted us to investigate if the pre-formed Zinc/d-bdnf complexes influence neurotrophic activity when compared to the unbound peptide. When analyzing the neurite outgrowth induced in differentiated SH-SY5Y cultures by d-bdnf (Figure 2B,C), we found that a pre-incubation with an equimolar amount of Zinc was able to abolish this effect (Figure 6A). In agreement with this, the increased expression of genes important for neuronal differentiation prompted by d-bdnf was almost inhibited (NTRK2, NEFM) or impaired (LAMC1) by pre-incubation of the peptide with Zinc ion (Figure 6B). A similar effect was found for the increased expression of axonal specification marker MAPT. The binding of the zinc ion with the N-terminal of the d-bdnf could probably hamper the receptor binding by modifying its tridimensional conformation.

## Conclusions

In this work, we tested the potential of a dimeric peptide constituted by 2 copies of the first 12 amino acids of the BDNF sequence held together by a cysteine bond to act as a neurotrophic mimetic peptide. Results indicate the ability of d-bdnf to promote phosphorylation of the TrkB receptor and downstream ERK and AKT effectors. This correlated with its ability to promote TrkB dimerization, although in the context of a dynamic monomer-dimer equilibrium, which resembles the one occurring with BDNF stimulation. This is supported, at least for the d-bdnf case, by the asymmetric interaction with two TrkB moieties as predicted from MD simulations. Whether this mode is conserved for the BDNF binding constitutes an exciting avenue for future investigations. Furthermore, d-bdnf was able to sustain neurite outgrowth and increase the expression of differentiation (NEFM, LAMC1) and polarization markers (MAP2, MAPT) demonstrating its neurotrophic activity.

In the synaptic cleft, the released free metal ions can reach up to micromolar concentrations and can modulate directly or indirectly the activity of several post-synaptic receptors or can bind different proteins and peptides modulating their activity.^10,12^ The d-bdnf activity revealed to be decreased by equimolar amounts of coordinated zinc, demonstrating the role of this metal ion in tuning the activity of this peptido-mimetic.

Overall, our data identify a novel BDNF mimetic peptide and uncover novel mechanistic details in its activation of the TrkB receptor. Although other investigations will be required to characterize in full its pharmacological properties, we report here the advantage of d-bdnf peptide of being a linear sequence, which can be straightforwardly implemented in higher-order constructs, for example, nanoparticles or other vectors. Its in-depth analysis may open the way to several possible future BDNF-based therapeutic applications.

## Materials and Methods

### Constructs, hBDNF Protein, and Peptido-Mimetic Preparation

The full-length human TrkB cDNA was cloned as C-terminal GFP fusion in pReceiver-M03plasmid (GeneCopoeia), to get the TrkB-eGFP construct. The farnesyl-eGFP construct (f-EGFP) has been previously described.^43^ The peptides HSDPARRGELSV-NH_2_ (m-bdnf) and the scrambled SRAGPHLRDVSE-NH_2_ (s-bdnf) were assembled using the solid phase peptide synthesis strategy on a Pioneer Peptide Synthesizer as previously described.^31^ The dimer peptide HSDPARRGELSVC-C-VSLEGRRAPDSH (d-bdnf) was purchased from CASLO (Lyngby, Denmark). The human proBDNF cDNA was cloned in the prokaryotic expression vector pET11a as previously reported for proNGF construct.^49,50^ The preparation of mature hBDNF has been recently described in detail.^32^

### Morphometric Analysis of SH-SY5Y Differentiation

At the end of the differentiation protocol, SH-SY5Y cells were observed using an optical microscope equipped with a 20× magnification objective. Typically, cells were seeded in 24 well-plates and at least 2 different wells were subjected to the same treatment in up to four independent replicas. For each well, we acquired 2–3 different fields to perform a morphometric analysis of SH-SY5Y differentiation. For each field, we quantified the neurite density as reported in Convertino et al.^32^ Briefly, we calculated the total neurite length per cell, that is, the ratio between the sum of the lengths of all neurites and the number of cell somas detected in each field using the Imagej software.

### N&B Analysis

5 h after transfection, TrkB-EGFP or f-EGFP transfected cells were trypsinized and transferred into glass-bottom WillCo chambers (at a density of 2–3 × 10^5^ cells per 22-mm-diameter dish). The next day, cells were serum starved in DMEM with 4.5 g/L glucose for at least 2 h before imaging. N&B measurements were performed by using an Olympus FluoView 1000 confocal equipped with pseudo-photon-counting detectors. Each acquisition consists of a time series of 100 frames, 256 × 256-pixels each, with a pixel size of 50 nm and a pixel dwell time of 4 μs. EGFP was excited with a 488-nm laser line, while its emission was collected in the 500–600-nm range. Low laser power was used at the sample to avoid photobleaching. The N&B analysis was performed using the SimFCS software V 2.0. Cells transiently transfected with f-EGFP were used to identify the appropriate experimental conditions (e.g., low expression level of the construct, laser power, and scanning speed) for the measurement of a membrane-diffusing protein. Additionally, f-EGFP was used as a monomeric reference. In total, 150 ng/mL BDNF and 10 μM d-bdnf treatments were added directly on the Willco plate, after which cells were observed within a 30 min time window. Measurements of the immobile fraction (the background signal) were performed to calibrate the S-factor of the microscope pseudo-photon-counting detector. As described in ref (35), *B* parameter represents the apparent brightness of the fluorescent species, and it is obtained for each pixel as the reciprocal of the ratio between the average fluorescence intensity and its associated variance. Pixels that contain immobile particles have *B* = 1; conversely, pixels containing mobile particles show *B* > 1.

### Docking Simulations

The starting coordinates of domain-5 of TrkB (TrkB-D5) were taken from the X-ray structure of TrkB-D5 bound to NGF (pdb code 1HCF).^51^ The former complex was used as template for the alignment of the main MD clusters of the d-bdnf prior to the docking to one chain of TrkB-D5. Docking simulations have been performed using the HADDOCK interface.^52^ All the d-bdnf residues were included as active residues for the Haddock docking, as well as T288 to F305; I334 to N338 belonging to TrkB-D5. The starting clusters of the first docking were used for the docking concerning the binding of one peptide with two TrkB chains. Structures underwent rigid body energy minimization, semirigid simulated annealing in torsion angle space, with a final clusterization of the results.

### HR-ESI-MS: Peptides–Zinc Interaction Studies

ZnSO_4_ hydrated was purchased from Merck and used without further purification. LC–MS grade water was used. All the solutions were freshly prepared before the incubation. Stock solutions (10^–2^ M) of peptides and ZnSO_4_ were freshly prepared in LC–MS grade water. Each peptide solution was diluted to a final concentration of 10^–6^ M and incubated for 2 h at 37 °C in the presence of a 100-fold excess of Zn. Aliquots of the various solutions were sampled after the incubation time and analyzed with or without the addition of 0.1% of formic acid before injection. Experimental parameters: spectra were acquired through direct infusion at 5 μL/min flow rate in a TripleTOF 5600^+^ mass spectrometer (Sciex, Framingham, MA, U.S.A.), equipped with a DuoSpray interface operating with an ESI probe in positive polarity. The ESI source parameters were optimized and were as follows: positive polarity, ionspray voltage floating 5500 V, temperature 30 °C, ion source gas 1 (GS1) 45 L/min; ion source gas 2 (GS2) 0 L/min; curtain gas (CUR) 15 L/min, declustering potential (DP) 100 V, collision energy (CE) 10 V. For acquisition, Analyst TF software 1.7.1 (Sciex) was used and deconvoluted spectra were obtained by using the Bio Tool Kit micro-application v.2.2 embedded in PeakView software v.2.2 (Sciex).

## Supporting Information Available

Additional experimental details, materials, and methods on preparation of hBDNF protein; SH-SY5Y and RenCell cell culture, transfection and differentiation; cell viability assay; phosphorylation of TrkB and downstream signaling effectors; RT-PCR analysis; peptide simulation details; potentiometric measurements; and statistical analysis (PDF)
