# Supplementary material for: Neurotrophic Activity and Its Modulation by Zinc Ion of a Dimeric Peptide Mimicking the Brain-Derived Neurotrophic Factor N-Terminal Region
Source: ACS Chem Neurosci. 2022 Nov 8;13(23):3453–63. doi: 10.1021/acschemneuro.2c00463 (PMC9732821; doi:10.1021/acschemneuro.2c00463)
Supplement: Supplementary file 1 — cn2c00463_si_001.pdf [file cn2c00463_si_001.pdf]

Supporting Information

Neurotrophic Activity and its modulation by Zinc ion of a Dimeric Peptide mimicking the Brain Derived Neurotrophic Factor N-terminal region

Lara Russo[1,$], Chiara Giacomelli[1,$], Mariagrazia Fortino[2], Tiziano Marzo[1], Gianmarco Ferri[3], Mariantonietta Calvello[4], Alessandro Viegi[4], Antonio Magrì[5], Alessandro Pratesi[6], Adriana Pietropaolo[2], Francesco Cardarelli[3], Claudia Martini[1], Enrico Rizzarelli[5,7], Laura Marchetti[1,*], Diego La Mendola[1,*], Maria Letizia Trincavelli[1]

[1] Dipartimento di Farmacia, Università di Pisa, 56127 Pisa, Italy

[2] Università di Catanzaro, 88100 Catanzaro, Italy

[3] Laboratorio NEST, Scuola Normale Superiore, 56127 Pisa, Italy

[4] Bio@SNS, Scuola Normale Superiore, 56126 Pisa, Italy

[5] Istituto di Cristallografia, Consiglio Nazionale delle Ricerche (CNR), 95126 Catania, Italy

[6] Dipartimento di Chimica e Chimica Industriale, Università di Pisa, 56124 Pisa, Italy

[7] Università degli Studi di Catania, 95124 Catania, Italy

[$] Equal Contributor

These authors contributed equally to the work

[*] Corresponding authors

laura.marchetti@unipi.it; diego.lamendola@unipi.it

Supplementary Figures

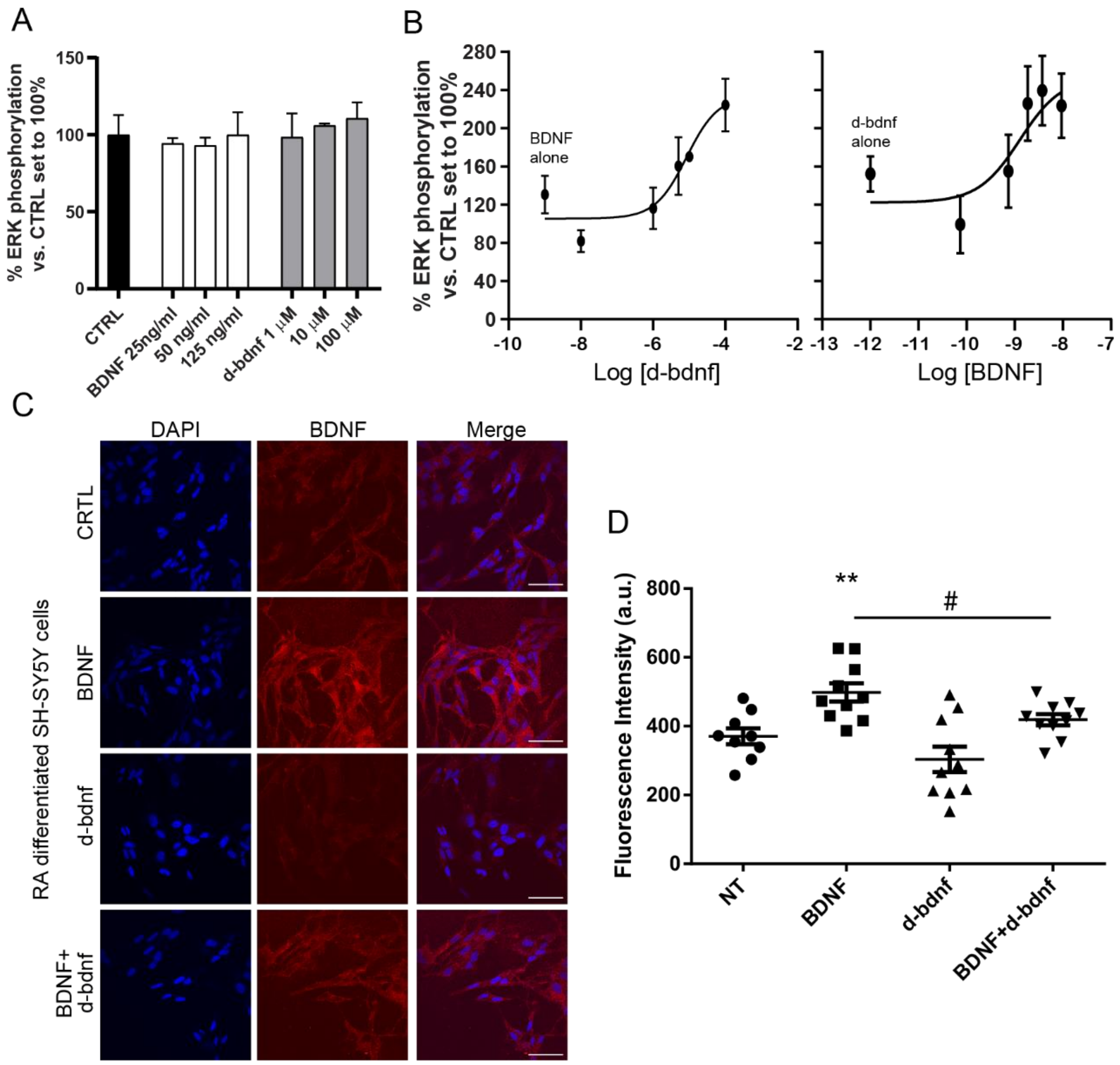

Figure S1. Evaluation of d-bdnf mechanism of action. A) ERK phosphorylation levels detected in HEK293T cells, after stimulation (10 min) with increasing concentrations of BDNF (white columns) or d-bdnf (grey columns) normalized to the unstimulated control (CTRL, black column) set to 100%. Data are reported as mean±SD and are not significantly different according to one-way ANOVA with Dunnett's multiple comparison test. **B)** ERK phosphorylation levels detected in differentiated SH-SY5Y cells, after stimulation (10 min) with a combination of a single concentration of BDNF (1 nM referred to monomeric form) with increasing concentrations (10 nM-100 µM) of d-bdnf (left); **B)** a combination of a single concentration of d-bdnf (10 µM) with increasing concentrations (0.074 nM – 10 nM) of BDNF (right). Phosphorylation levels were normalized to the cell number and reported as percentage versus the ctrl (untreated). Data ± SEM were pooled from 2 independent replicas. **C)** Representative confocal fluorescence images of SH-SY5Y cells differentiated with retinoic acid (RA) and treated acutely with BDNF, d-bdnf or a combination of the two. Cells were then subjected to immunofluorescence using α-BDNF antibody and stained with DAPI. Scale bar: 50 µm.**D**) Scatter dot plot of the mean ± SEM fluorescence intensity of the analyzed fields (n_{fields}) for the different samples (NT: n_{fields}=9 and total n_{cells}= 285; BDNF: n_{fields}=10 and total n_{cells}= 297; d-bdnf: n_{fields}=10 and total n_{cells}= 255 cells; BDNF+d-bdnf: n_{fields}=10 and total n_{cells}= 253 cells; **p<0.01 according to

one-way ANOVA with Dunnett’s multiple comparison test, # p<0.05 versus BDNF, according to one-way ANOVA with Sidak’s multiple comparison test).

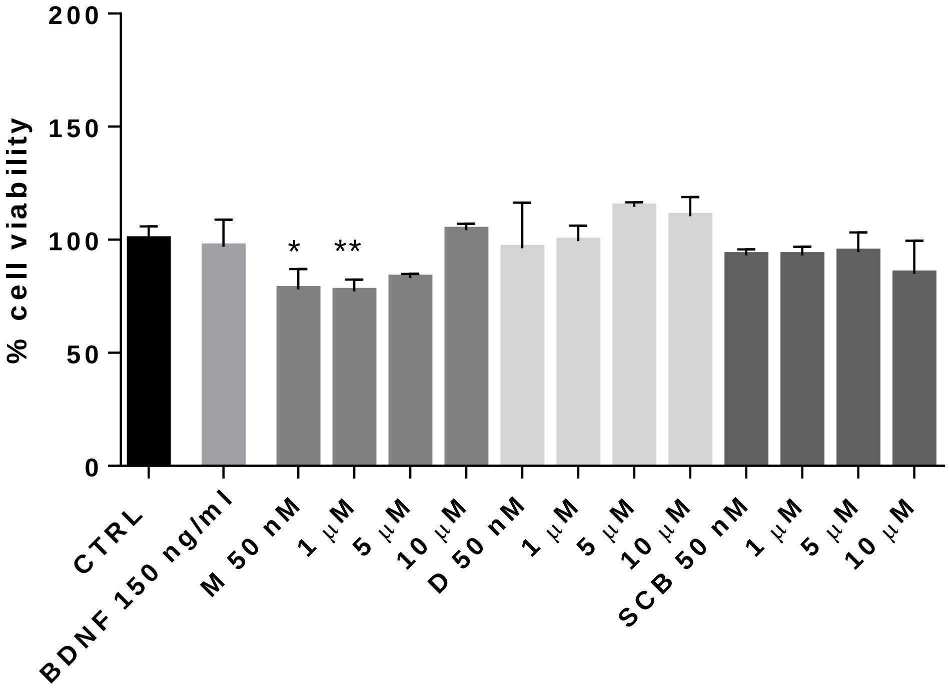

Figure S2. MTS assay for BDNF and its mimetic peptides. Cell viability assay of RA-differentiated SH-SY5Y cells treated for three days with 150 ng/ml BDNF of with different concentrations of m-, d- or s-bdnf peptides (M, D and S, respectively). ** $p<0.01$, *$p<0.05$, according to one-way ANOVA with Dunnett’s multiple comparison test).

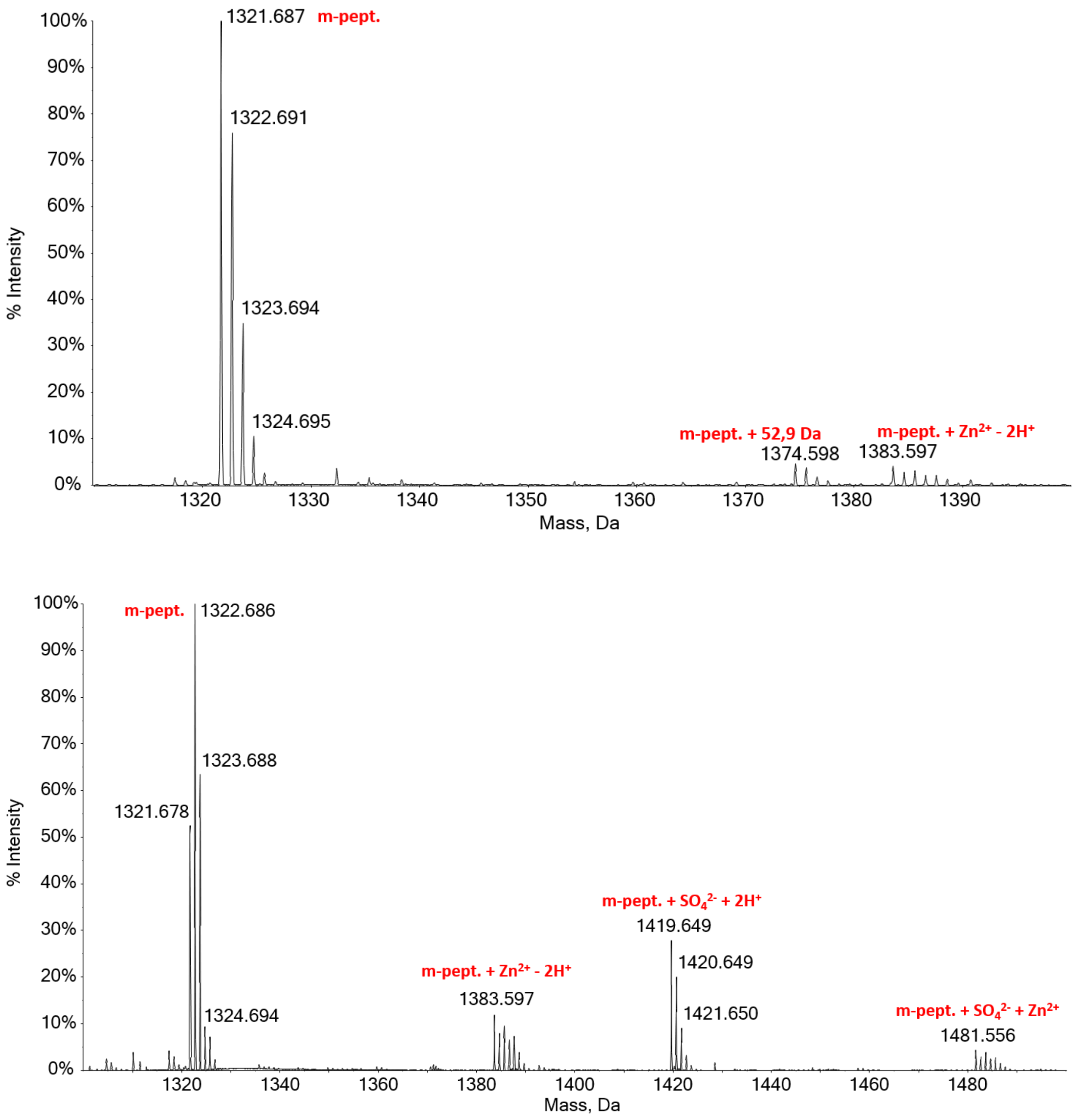

Figure S3. Deconvoluted ESI mass spectra of 10^{-6} M m-bdnf solutions incubated in water 2 h, 37° C in presence of a 100-fold excess of Zn^{2+}. 0.1 % of formic acid was added (top) or not (bottom) just before the infusion.

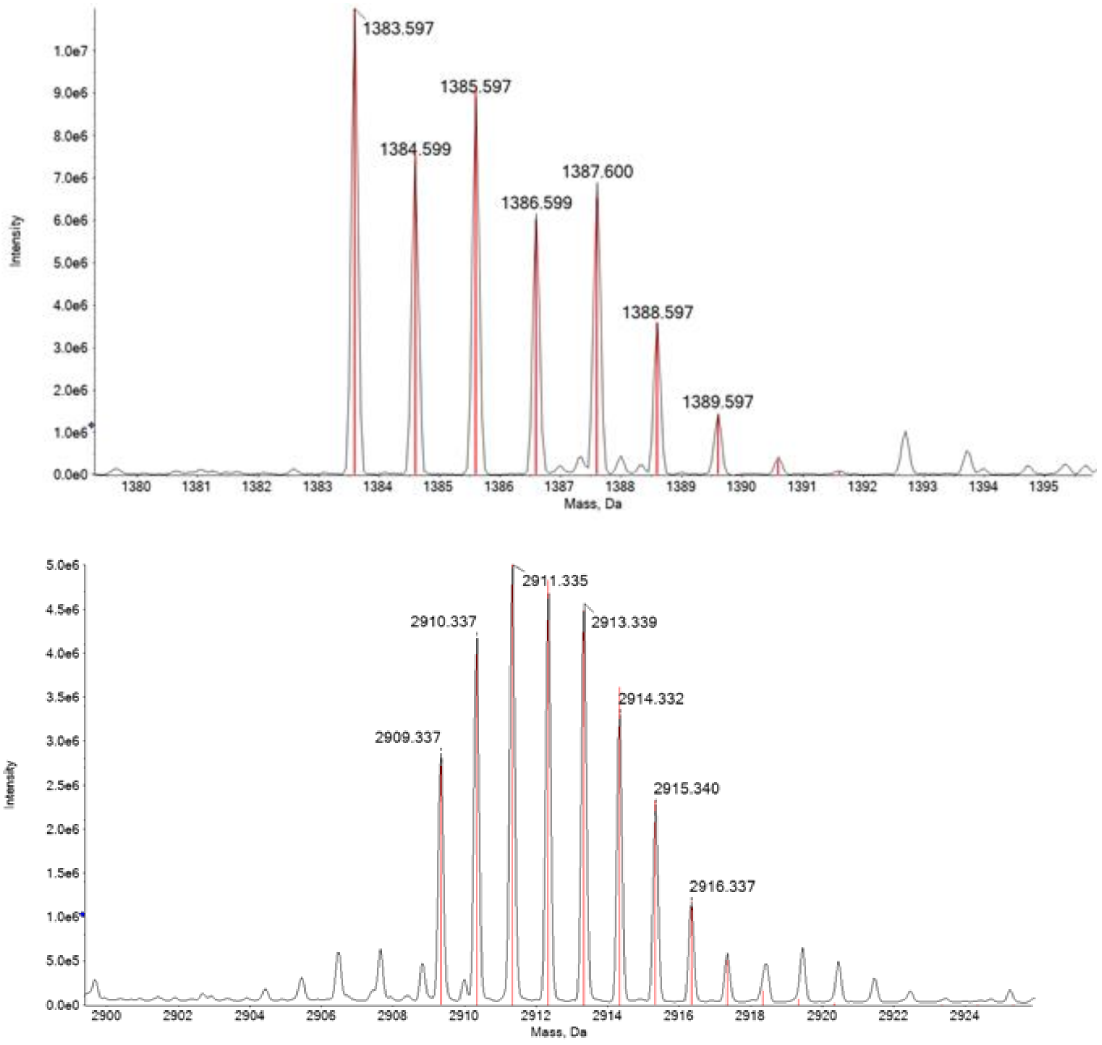

Figure S4. Simulation of the isotope pattern and error calculation. Top: High-resolution ESI mass spectrum of monopeptide-Zn^{2+} adduct, 10^{-6} M in H_2O. Experimental isotopic distribution for $C_{54}H_{89}N_{21}O_{18}Zn$ (black line) vs the theoretical one (red lines). Deconvoluted measured mass = 1383.59735 Da; theoretical mass = 1383.59859 Da; mass error = -0.9 ppm. Bottom: High-resolution ESI mass spectrum of dipeptide-Zn^{2+} adduct, 10^{-6} M in H_2O. Experimental isotopic distribution for $C_{114}H_{192}N_{46}O_{36}S_2$
Zn (black line) vs the theoretical one (red lines). Deconvoluted measured mass = 2909.33710 Da; theoretical mass = 2909.33403 Da; mass error = 1.0 ppm.

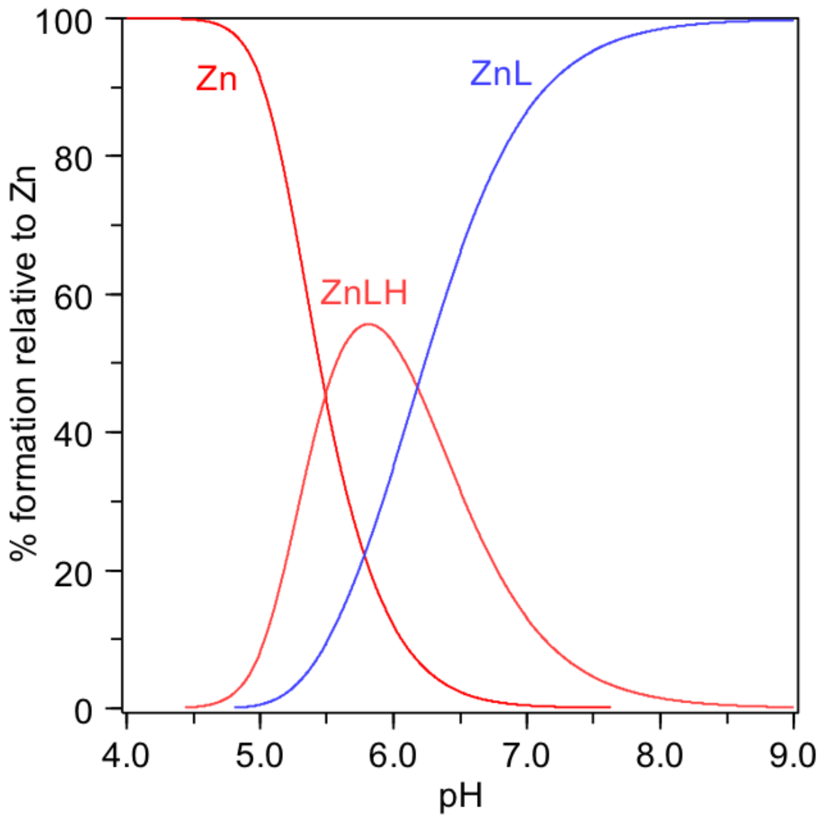

Figure S5. Species distribution diagram for Zn^{2+} complexes with d-bdnf. Charges are omitted for clarity. [L] = 1×10^{-3} mol dm^{-3}; M/L molar ratio of 1:1.

Supplementary Tables

Table S1. Main H-bonds observed in the binding of TrkB/d-bdnf, indicating the amino acid residues participating to the non-covalent interactions.

**TrkB-BDNF binding pose a)**	
TrkB	peptide
Thr288:OG1	Arg21:HH21
Thr290:HG1	Glu23:OE2
Ser294:O	Ser11:HG
Asp298:OD2	Arg7:HH1
Lys308:HZ1	Glu23:OE1
Asn338:OD1	Ser2:HG
**TrkB-BDNF binding pose b)**	
TrkB	peptide
Thr290:O	Arg7:HH12
Glu293:OE2	Ala5:HN
Glu293:OE1	Ser2:HG
Asp298:OD1	Arg20:HH21
His299:ND1	His15:HE2
His300:HE2	Asp17:OD1
Asn338:HD22	Ser11:O
His343:O	Glu23:HN
Gln347:OE2	Arg21:HH12

Supplementary Table S2. Main H-bonds observed in the binding of TrkB/d-bdnf/TrkB, indicating the aminoacid residues participating to the non-covalent interactions.

**TrkB1- d-bdnf- TrkB2 binding pose a)**			
TrkB1	peptide	TrkB2	peptide
His299:ND1	Asp17:HN	Asp298:OD2	His15:HE2
Thr306:OG1	His1:HE2	His300:HE2	Leu10:O
Lys308:HZ2	His1:O	Lys333:HZ2	Glu23:OE1
Gln347:OE1	Arg20:HH22	His335:HD1	Ala19:O
		Val336:HN	Arg21:O
		Glu341:OE2	Arg21:HH11
		His343:HD1	Pro18:O
**TrkB1- BDNF- TrkB2 binding pose b)**			
TrkB1	peptide	TrkB2	peptide
Ser294:O	Ser16:HG	Thr296:OG1	Gly22:HN
Asp298:OD	His15:HE2	His299:HD1	Asp3:OD2
His299:HN	Ser25:O	Lys308:HZ2	Val26:O
Lys333:HZ3	Ser11:O	His335:HD1	Glu9:OE2

His335:HE2<br>Thr337:O<br>Asn338:O	Ala19:O<br>Arg7:HH11<br>Arg7:HH12	His335:HE2<br>His335:HE2<br>Val336:HN<br>His343:HD1	His:ND1<br>Ser25:O<br>Glu23:OE1<br>Glu23:O

Supplementary Table S3. Protonation and complex stability constant values of Zn^{2+} bound to d-bdnf peptide.

Species			
LH	logβ	logk	
11	7.59	7.59	NH_2
12	14.75	7.16	NH_2
13	20.71	5.96	His
14	26.03	5.32	His
15	30.69	4.66	COO
16	34.90	4.20	COO
17	38.44	3.55	COO
18	41.75	3.31	COO

ZnLH	logβ	logk	
111	13.30 (2)	-	2 His, NH_2
110	7.15 (3)	6.18	2 His, 2 NH_2

Supplementary Table S4 List of primer sequences

Gene	Coded protein	Primer Sequences	Amplicon Length
MAPT	*Microtubule-associated protein tau*	F: GATTGGGTCCCTGGACAATA<br>R: GTGGTCTGTCTTGGCTTTGG	106 bp
MAP2	*Microtubule-associated protein 2*	F: CAGAGGAGGTGTCTGCAAGGA<br>R: GCAGAGCTGCAGTCTGGTCTTT	108 bp
LAMC1	*Laminin subunit gamma-1*	F: GAGAAAACAAGATGACGCTG<br>R: TTTAGGGTGCCTTCAATCTC	201 bp
NTRK2	*TrkB*	F: GGCAATCCATTTACATGCTC<br>R: TGCCACACTACAGGATAATG	225 bp
GAP43	*Neuromodulin*	F: TTACCTTGCCTGGGAGGCTT<br>R: AATCTTTTGGTCGTCATCA	124 bp
GAPDH	*Glyceraldehyde-3-phosphate dehydrogenase*	F: GAGAAGTATGACAACAGCCT<br>R: CCTTCCACGATACCAAAGTT	107 bp

Supplementary materials and methods

Preparation of hBDNF protein

The preparation of mature hBDNF has been recently described in detail [1]. Briefly, BL21(DE3) E. coli were transformed with hproBDNF construct in pET11a plasmid, and protein expression induced with 1 mM of isopropyl-b-thio-galactoside (IPTG). After 5 hours bacteria were collected by centrifugation, and the pellet resuspended with Lysis Buffer (10 mM TRIS HCl pH 8, 1 mM EDTA and 1 mg/ml lysozyme), incubated at room temperature for 1 h, sonicated on ice, and incubated again with a 3 mM MgCl2 and 50 ug/ml DNaseI solution at room temperature for 30 minutes. hproBDNF was recovered from isolation and solubilization of inclusion bodies in denaturing conditions [1], and further refolded by drop by drop addition in 100 ml Refolding buffer (1 M Arginin, 100 mM TRIS HCl pH 9.3, 5 mM EDTA pH 8, 1mM GSSG and 5 mM GSH). Every hour 50 µg/ml of denatured sample were added to the buffer under vigorous stirring at 4 °C. After 16-48 hours the sample was dialyzed at 4 °C in IEX-A (50 mM Na phosphate pH 7, 1 mM EDTA) for 12 hours. The dialyzed sample containing proBDNF was filtered using a 0.22 µm filter and purified by FPLC. The sample was loaded on a HiLoad 16/10 SP Sepharose High Performance (~20 ml - GE Healthcare) equilibrated with IEX-A buffer using a liquid chromatography system (ÄKTA™ - FPLC). The protein was eluted with linear gradient from 0 to 100% of IEX-B (50 mM Na phosphate pH 7, 1 mM EDTA and 1M NaCl) at 1ml/min flow in 6 column volume (CV) and 2ml fractions collected. The ones corresponding to the UV (280nm) FPLC peak containing hproBDNF were pooled and dialyzed against 100 mM Hepes pH 7.5, 1 mM CaCl2. The mature hBDNF protein was obtained from the purified hproBDNF after digestion with previously prepared His-tagged human furin (1µg enzyme : 20 µg of propeptide), at 30 °C for 2 h. The expression and purification of recombinant His-tagged human furin is elsewhere reported [1]. The hBDNF was purified by ion exchange chromatography with the same protocol used for the prohBDNF already described. The fractions collected were pooled and dialyzed against 1 L of Storage buffer (50 mM Na phosphate pH 7, 150 mM NaCl) for 16 hours at 4 °C. The dialyzed protein was concentrated up to 1mg/ml using an Amicon ultrafiltration membrane with 10 kDa cut-off (Merck-Millipore) and stored at - 80 °C. The purity and the correct molecular weight of the protein was monitored by SDS-PAGE and MS analyses.

Peptide synthesis and purification

The peptides HSDPARRGELSV-NH2 (m-bdnf) and the scrambled sequence SRAGPHLRDVSE-NH_2 (s-bdnf) were assembled using the solid phase peptide synthesis strategy on a Pioneer Peptide

Synthesizer. All amino acid residues were added according to the TBTU/HOBT/ DIEA activation method for Fmoc chemistry on a NovaSyn-TGR resin (loading 0.18mmolg 1, 0.33mmol scale synthesis). Removal of Fmoc protection was achieved by means of a 20% piperidine solution in DMF. The peptides were purified by means of a preparative reversed-phase high-performance liquid chromatography (RP-HPLC). Purification was performed on a Varian PrepStar 200 model SD-1 chromatography system equipped with a Prostar photodiode array detector with detection measured at 222 nm. They were eluted with a gradient of a solvent A (0.1% TFA in water) and B (0.1% TFA in acetonitrile) on a Vydac C18 250 x 22 mm (300 pore size, 10–15 mm particle size) column, at a flow rate of 0.2 mL min^{-1}. The peptides were eluted according to the following protocol: from 0 to 5min isocratic gradient in 0% B, then linear gradient from 0 to 15 % B over 25 min, finally isocratic gradient in 15% B from 25 to 40 min. The peptides were characterized by means of electron spray ionization mass spectrometry (ESI-MS).

HSDPARRGELSV-NH2 (m-bdnf): Rt = 25.2 min. ESI-MS: calculated for C54H91N21O18: 1322.4 [M]; found: 1323.2 $[M + H]^{+}$; 662.1 $[M + 2H]^{2+}$]. [2]

SRAGPHLRDVSE-NH_2 (s-bdnf): Rt = 27.4 min. ESI-MS: calculated for C54H91N21O18: 1322.4 [M]; found: 1323.2 $[M + H]^{+}$; 662.1 $[M + 2H]^{2+}$].

Cell culture, transfection and differentiation

Human neuroblastoma SH-SY5Y cells (a kind gift of Prof. M. Canossa, University of Trento) were maintained in a humidified atmosphere at 37 °C, 5% CO_2 in Dulbecco's modified eagle medium with nutrient mixture F12 (DMEM-F12, Corning) supplemented with 10% fetal bovine serum (FBS, Corning) and 1% penicillin/streptomycin (Corning). Human HEK 293 cells (ATCC, CRL-11268) were maintained in a humidified atmosphere at 37 °C, 5% CO_2 in Dulbecco's modified eagle medium (DMEM, Corning) supplemented with 4.5 g/L glucose (Sigma Alrich), 10% fetal bovine serum (FBS, Corning) and 1% penicillin/streptomycin (Corning). Transfections of undifferentiated cells were performed by using the LipofectamineTM 2000 reagent (Thermo Fisher Scientific), according to the manufacturer's instructions. For cell differentiation, sub-confluent cells (~$4x10^3$ cells per cm^2) were seeded onto the bare substrates and 24-48 hours later, they were first subjected to a 5-days treatment with 10 µM retinoic acid (RA, Sigma-Aldrich) in complete DMEM-F12, and then to a 3 days-treatment with 150 ng/ml hBDNF or different concentrations of BDNF peptide-mimetics in serum-free DMEM-F12. Every 2-3 days, 2/3 of the medium was renewed. As reference controls, we used cells at the same confluency but either incubated for 3 days in serum-free DMEM-F12 after 5 days in complete medium (differentiation ctrl), or cells subjected to the 5-days RA treatment, and then

incubated for 3 days in serum-free DMEM-F12 (morphometric ctrl). Alternatively, in order to investigate genes involved in the neuronal polarization during BDNF treatment, we used cells subjected to the 5-days RA treatment only as a reference (polarization ctrl).

Cell viability assay

SH-SY5Y were seeded at a density of 3.000 cell/well in 96 multiwell and differentiated as describe above. The viability of differentiated cells, in response to the various treatments, was assessed using an MTS assay (CellTiter 96 AQueous One Solution Cell Proliferation Assay kit; Promega) according to the manufacturer's instructions. The absorbance of formazan at 490 nm was measured in a colorimetric assay with the EnSightTM multimode plate reader (Perkin Elmer). Data were reported as percentage versus the control set to 100%.

Phosphorylation of TrkB and downstream signaling effectors

TrkB phosphorylation was monitored after TrkB-eGFP construct immunoprecipitation, similarly to what previously reported for TrkA phosphorylation [3]. Briefly, 48 h after transfection, SH-SY5Y cells were starved for two hours in serum-free DMEM/F-12 medium. Then they were incubated for 10 minutes at 37°C in PBS, either in absence or presence of 150 ng/ml BDNF or 10 µM of the m-, d-, or s- BDNF peptide-mimetics. Cells were finally washed once with PBS and finally lysed in RIPA buffer supplemented with phosphatase (5mM NaF, 2 mM sodium orthovanadate, Sigma Aldrich) and protease inhibitors (4 ug/ml aprotinin, 0.1 mg/ml Phenylmethylsulfonyl fluoride, Sigma Aldrich). Total cell extracts (150-300 µg) were incubated with 1 µl of α-GFP (ab290, Abcam) antibody overnight at 4°C under rotary shaking. The mixture of antibody and lysates were then incubated 30 minutes at room temperature with 50 ul Dynabeads protein G (Termo Fisher Scientific). The resulting complexes in the mixture were then magnetically isolated and beads washed three times with PBS with 0.002% Tween-20 (PBST), and then boiled in 2X Laemmli loading buffer at 95 °C for 10 minutes. Samples were loaded on a 4-20 % pre-cast gradient gel (Criterion TGX Stain-Free, Biorad) and electrotransferred on a PVDF membrane (Trans-Blot Turbo, Biorad). Gels were activated for 2.5 min and imaged, before and after transfer, using the ChemiDoc MP imaging system (Bio-Rad). Membranes were blocked for 1 hour at RT with Tris Buffered Saline+ 0.05% Tween-20 (TBST) supplemented with 5% w/v BSA (Sigma). After blocking, membranes were blotted overnight at 4°C with anti-phosphotyrosine (05-321 Millipore, dilution 1:1000) or anti-TrkB (sc-136990, Santa Cruz Biotechnolgy; dilution 1:100) antibody in blocking solution. The primary antibodies were detected by using an anti-mouse secondary antibody horseradish peroxidase (HRP)-conjugated (Sigma, diluted 1:5000). Blotted membranes were incubated with Clarity Western ECL substrate or Clarity

Max Western ECL substrate (Biorad) and imaged with ChemiDoc MP imaging system (Bio-Rad). Densitometric analysis of the obtained bands was done with ImageJ software (https://imagej.nih.gov/ij/) or ImageLab software (Bio-Rad).

ERK and AKT phosphorylation were assessed in a 96-well plate format, performing an in cell ELISA assay as previously described [4]. SH-SY5Y or HEK 293T cells were seeded at a density of 5.000 and 80.000 cell/well in 96 multiwell respectively, and the former were differentiated as describe above. Cells were treated as described for TrkB phosphorylation or reported in the graphs, then washed with 0.1% Triton X-100 in PBS and fixed with 4% paraformaldehyde. Cells were then permeabilized (1% BSA; 0.1% Triton X-100 in PBS) for 1 hour, at the end, cells were incubated overnight at 4°C with anti-phospho ERK (1:500, sc-7383, Santa Cruz) or phosphor AKT (1:500, sc-16646-R-, Santa Cruz) antibodies. Next day, after the incubation with HRP-conjugated secondary antibody (anti-mouse # 31430, Invitrogen; anti-rabbit A0545, Sigma-Aldrich) the TMB substrate was added and the absorbance was read at 450 nm. Cells were incubated with only the secondary antibody to obtain the aspecific binding (Blanks). The absorbances were normalized to the relative number of cells in each well that was determined using Crystal Violet staining.

SH-SY5Y Immunofluorescence analysis

SH-SY5Y cells were seeded on a 24-well plate (5.000 cell/well) and differentiated with retinoic acid as describe above. Cells were treated for 60’ in an unsupplemented medium with BDNF (150 ng/ml) or d-bdnf (10 µM) alone or in combination. Samples were then fixed (4% PFA, 5% sucrose/PBS, 15 min) and subjected to a non-permeabilizing immunofluorescence assay. Briefly, cells were incubated with the blocking solution (5% BSA/PBS) for 1 h at room temperature and incubated overnight at 4 °C with chicken anti-BDNF (1:100, G164A Promega) antibody diluted in 2.5% BSA/PBS. The next morning samples were incubated with an Alexa568-conjugated anti-chicken secondary antibody (1:500, Thermofisher) in 2.5% BSA/PBS for 2h at room temperature. Coverslips were mounted with Fluoroshield-DAPI (Sigma-Aldrich) and imaged with a laser scanning confocal microscope (Nikon A1, Eclipse Ti), using a 60× oil objective (NA 1.40) and pinhole set to 1 AU. Images were acquired at 1024 pixel × 1024 pixel resolution using a 405 nm laser (emission window 400-500 nm) and a 561 nm laser (emission window 545-645 nm). BDNF fluorescence intensity was analysed using FIJI ImageJ software. Briefly, the background intensity was subtracted from all red channel fields and an image threshold was applied to calculate the mean fluorescence intensity of the cells only. Results shown constitute the pool of fields stemming from two replicates.

ReNCell cell culture, differentiation and immunofluorescence analysis

ReNcell VM Human Neural Progenitor Cell Line (Cat. # SCC008, Millipore) were maintained in a humidified atmosphere at 37 °C, 5% CO_2 ReNcell NSC Maintenance Medium (Millipore Cat. No. SCM005) containing 20 ng/mL bFGF-2 and 20 ng/mL EGF. Cells were cultured in T75 flasks coated with laminin (20 µg/mL). The coating solution was incubated in a 37 °C, 5% CO_2 incubator for at least 4 hours and then washed with PBS. For differentiation, cells were seeded at a density of 30.000 cell/cm^2 onto glass coverslips after the laminin coating in complete medium. The next day, differentiation was initiated by removing the medium from each well and replacing with fresh ReNcell NSC Maintenance Medium without bFGF-2 and EGF. Cells were treated with BDNF (50 ng/ml) or d-bdnf (1 µM) in differentiation medium for four days. At the end, treated samples were fixed (4% PFA, 5% sucrose/PBS) and subjected to the immunofluorescence assay for detection of β3 tubulin. Briefly, cells were incubated with the blocking solution (5% BSA/PBS) for 1 h at room temperature, permeabilized 10’ with 2.5% BSA/PBS 0.1% Triton X-100, washed in PBS and incubated overnight at 4 °C with mouse anti-β3tubulin (1:200, TU20 Cell Signaling) antibody. The next morning samples were incubated with an Alexa488-conjugated anti-mouse secondary antibody (1:100, Thermofisher) in 2.5% BSA/PBS for 2h at room temperature. Coverslips were then mounted with Fluoroshield-DAPI (Sigma-Aldrich) and imaged with the laser scanning confocal microscope as described in the previous section, using a 20× air objective (NA 0.75) and pinhole set to 1 AU. Images were acquired at 1024 pixel × 1024 pixel resolution using a 405 nm laser (emission window 400-500 nm) and a 488 nm laser (emission window 475-575 nm). The acquired images were analysed using FIJI ImageJ software. β3Tubulin positive cells were identified in each field by visual inspection and normalised on the total cells counted in the DAPI channel.

RT-PCR analysis of SH-SY5Y differentiation

At the end of each differentiation treatment and relative controls, cells were collected, and total RNA was extracted using the Rneasy Mini Kit (Qiagen). The RNA concentration and purity were checked at the Nanodrop instrument (Thermofisher). cDNA synthesis was performed with 1 µg of RNA using i-Script cDNA synthesis kit (BioRad). Real-time PCR reactions consisted of 10 µL of SsoAdvanced™ Universal SYBR® Green Supermix - Bio-Rad, 0.5 µL of both 10 µM forward and reverse primers, 5 µL of cDNA (50 ng), and 4 µL of H_2O. The primer sequences and the product size are listed in Table S4. HPRT1 and NEFM primers were purchased by Biorad (qHsaCID0016375 and qHsaCED0043021, Bio-Rad). The mRNA levels for each sample were normalized against GAPDH and HPRT1 mRNA levels, and the relative expression was calculated by using the methods of the ΔΔCt value.

Peptide Simulation Details

The initial coordinates of d-bdnf connected through a disulfide bridge have been obtained using CHIMERA software [5]. Those coordinates have been energy minimized through 500000 Steepest Descent followed by 28 ns of equilibration with standard molecular dynamics using the amber99SB force field [6] and the TIP3P potential [7] for water solvent molecules, within GROMACS MD code - version 2020.2 [8].

Parallel Tempering Molecular Dynamic (PT-MD) simulations have been subsequently performed using the GROMACS MD code - version 2020.2 [8] in the NVT ensemble. All simulation data have been collected over 36 different temperatures (302.60 K, 305.22 K, 307.86 K, 310.52 K, 313.19 K, 315.84 K, 321.28 K, 324.01 K, 329.54 K, 335.14 K, 340.80 K, 346.55 K, 352.36 K, 358.24 K, 364.20 K, 370.24 K, 376.36 K, 382.56 K, 388.83 K, 395.18 K, 401.61 K, 408.13 K, 414.73 K, 421.42 K, 428.26 K, 435.12 K, 442.06 K, 449.09 K, 456.22 K, 463.44 K, 470.76 K, 478.17 K, 485.69 K, 493.29 K, 497.13 K, 501.00 K).

The Particle Mesh Ewald method [9] has been used to calculate electrostatic interactions. The time-step has been set to 2 fs. The LINCS algorithm [10] has been applied to fix all bonds lengths. For the Lennard-Jones and electrostatic interactions, periodic boundary conditions have been applied with a cutoff of 1.2 nm. All replicas were simulated in NVT ensemble using a stochastic thermostat [11] with a coupling time of 0.1 ps. A thermostat that yields the correct energy fluctuations of the canonical ensemble is crucial in enhanced sampling simulations [12–14]. Exchanges were attempted every 0.1 ps. The method of Daura and Van Gunsteren [15] was used in post-processing stage to cluster the resulting trajectories, with a cutoff of 3.5 Å calculated on the backbone atoms as implemented in the clustering utility provided in the GROMACS package [8].

The first 5 clusters have been chosen as the most representative ones with a percentage of representativity of 23,1%, 21,3%, 19,7%, 18,5%, 17.4%, respectively. The RMSD values calculated for all the clusters against the most representative one are 4.05 Å for cluster 2, 5.417 Å for cluster 3, 3.370 Å for cluster 4 and 3.37 Å for cluster 5.

The d-bdnf coordinates coordinating Zn(II) metal ion have been energy minimized by using the Steepest Descent algorithm and equilibrated through 26 ns of standard molecular dynamic simulation by using amber99SB as force field [6] in conjunction with Zn-parameter selected from Zinc Amber Force Field (ZAFF) [16] . The TIP3P potential [7] has been used for water solvent molecules in a simulation box of 6.0 x 6.0 x 6.0 nm3. The MD simulation has been performed by using GROMACS MD code - version 2020.2 [8].

CD measurements

Far-UV CD spectra were obtained at 298 K under a constant flow of nitrogen by using a Jasco model 810 spectropolarimeter. Measurements were carried out in water by using 1 cm path length cuvettes in the range λ=200–260 nm. All solutions were freshly prepared with doubly distilled water. Far-UV CD spectra were acquired by using Zn2+ and peptide concentrations ranging from $2.0x10^{-6}$ to $2.0x10^{-5}$ M.

Potentiometric measurements

Potentiometric titrations were carried out on a Titrando 905 automatic, using a combined glass-Ag/AgCl electrode (Metrohm, Switzerland). Solutions (2.0 mL) were thermostated at 298 K and in argon atmosphere. The ionic strength was 0.1 M in KNO3. A 0.1 M solution of KOH was used to titrate solutions containing either the peptides only (protonation constants) or the peptides with Zn2+ (complexation constants). The peptide concentrations range used was 1-2 mM in both series of experiments. At least three independent titrations were performed. Starting pH value was adjusted to 2.4 by adding HNO3 0.2 M. The titrations were carried out up to pH 8.5. Metal ion/ligand ratios between 0.9:1 and 2.2:1 were employed. To avoid systematic errors and verify reproducibility, the EMF values of each experiment were recorded at different time intervals. The data were analyzed by using HYPERQUAD 2003 program [17]. Species distribution as a function of pH was obtained by using Hyss program [18].

Statistical Analysis

The Graph-Pad Prism program (GraphPad Software Inc., San Diego, CA, United States) was used for data analysis and graphic presentation. Statistical analysis was performed by ordinary one-way analysis of variance (ANOVA) with one way Bonferroni post-hoc, or t-Test, with details indicated in the relative figure captions. All data values were reported as the mean of the values obtained in at least two independent experiments. $P \leq 0.05$ was considered statistically significant.

Supplementary References

(1) Convertino, D.; Mishra, N.; Marchetti, L.; Calvello, M.; Viegi, A.; Cattaneo, A.; Fabbri, F.; Coletti, C. Effect of Chemical Vapor Deposition WS2 on Viability and Differentiation of SH-SY5Y Cells. *Front. Neurosci.* **2020**, *14*, 592502. https://doi.org/10.3389/fnins.2020.592502.

(2) Travaglia, A.; La Mendola, D.; Magrì, A.; Nicoletti, V. G.; Pietropaolo, A.; Rizzarelli, E. Copper, BDNF and Its N-Terminal Domain: Inorganic Features and Biological Perspectives.

Chem. - A Eur. J. **2012**, *18* (49), 15618-31. https://doi.org/10.1002/chem.201202775.

(3) Amodeo, R.; Nifosì, R.; Giacomelli, C.; Ravelli, C.; La Rosa, L.; Callegari, A.; Trincavelli, M. L.; Mitola, S.; Luin, S.; Marchetti, L. Molecular Insight on the Altered Membrane Trafficking of TrkA Kinase Dead Mutants. *Biochim. Biophys. Acta - Mol. Cell Res.* **2020**, *1867* (2), 118614. https://doi.org/10.1016/j.bbamcr.2019.118614.

(4) Giacomelli, C.; Trincavelli, M. L.; Satriano, C.; Hansson, Ö.; La Mendola, D.; Rizzarelli, E.; Martini, C. Copper (II) Ions Modulate Angiogenin Activity in Human Endothelial Cells. *Int. J. Biochem. Cell Biol.* **2015**, *60*, 185-96. https://doi.org/10.1016/j.biocel.2015.01.005.

(5) Pettersen, E. F.; Goddard, T. D.; Huang, C. C.; Couch, G. S.; Greenblatt, D. M.; Meng, E. C.; Ferrin, T. E. UCSF Chimera - A Visualization System for Exploratory Research and Analysis. *J. Comput. Chem.* **2004**, *25* (13), 1605–1612. https://doi.org/10.1002/jcc.20084.

(6) Ivani, I.; Dans, P. D.; Noy, A.; Pérez, A.; Faustino, I. Europe PMC Funders Group PARMBSC1: a refined force-field for dna simulations. *Nat. Methods* **2016**, *13* (1), 55–58. https://doi.org/10.1038/nmeth.3658.PARMBSC1.

(7) Jorgensen, W. L.; Chandrasekhar, J.; Madura, J. D.; Impey, R. W.; Klein, M. L. Comparison of Simple Potential Functions for Simulating Liquid Water. *J. Chem. Phys.* **1983**, *79* (2), 926–935. https://doi.org/10.1063/1.445869.

(8) Lindahl, Erik; Abraham, Mark J; Hess, Berk; van der Spoel, D. GROMACS 2020.2 Source Code (Version 2020.2). **2020**. https://doi.org/10.5281/zenodo.3773801.

(9) Essman, U; Perera, L; Berkowitz, M. L. A Smooth Particle Mesh Ewald Method. *J. Chem. Phys* **1995**, *103*, 8577. https://doi.org/https://doi.org/10.1063/1.470117.

(10) Hess, B. P-LINCS: A Parallel Linear Constraint Solver for Molecular Simulation. *J. Chem. Theory Comput.* **2008**, *4* (1), 116–122. https://doi.org/10.1021/ct700200b.

(11) Bussi, G.; Donadio, D.; Parrinello, M. Canonical Sampling through Velocity Rescaling. *J. Chem. Phys.* **2007**, *126* (1). https://doi.org/10.1063/1.2408420.

(12) Losasso, V.; Pietropaolo, A.; Zannoni, C.; Gustincich, S.; Carloni, P. Structural Role of Compensatory Amino Acid Replacements in the α-Synuclein Protein. *Biochemistry* **2011**, *50* (32), 6994–7001. https://doi.org/10.1021/bi2007564.

(13) Pietropaolo, Adriana; Branduardi, Davide; Bonomi, Massimiliano; Parrinello, M. A Chirality-Based Metrics for Free-Energy Calculations in Biomolecular Systems. *J. Comput. Chem.* **2011**, *32*, 2627–2637. https://doi.org/DOI 10.1002/jcc.21842.

(14) Rosta, E.; Buchete, N. V.; Hummer, G. Thermostat Artifacts in Replica Exchange Molecular Dynamics Simulations. *J. Chem. Theory Comput.* **2009**, *5* (5), 1393–1399. https://doi.org/10.1021/ct800557h.

(15) Daura, X.; Gademann, K.; Jaun, B.; Seebach, D.; Van Gunsteren, W. F.; Mark, A. E. Peptide Folding: When Simulation Meets Experiment. *Angew. Chemie - Int. Ed.* **1999**, *38* (1–2), 236–240. https://doi.org/10.1002/(sici)1521-3773(19990115)38:1/2<236::aid-anie236>3.0.co;2-m.

(16) Peters, M. B.; Yang, Y.; Wang, B.; Füsti-Molnár, L.; Weaver, M. N.; Merz, K. M. Structural Survey of Zinc-Containing Proteins and Development of the Zinc AMBER Force Field (ZAFF). *J. Chem. Theory Comput.* **2010**, *6* (9), 2935–2947. https://doi.org/10.1021/ct1002626.

(17) Gans, P.; Sabatini, A.; Vacca, A. Talanta Suite of Programs. *Talanta* **1996**, *43*, 1739–1753.

(18) Alderighi, L.; Gans, P.; Ienco, A.; Peters, D.; Sabatini, A.; Vacca, A. Hyperquad Simulation and Speciation (HySS): A Utility Program for the Investigation of Equilibria Involving Soluble and Partially Soluble Species. *Coord. Chem. Rev.* **1999**, *184* (1), 311–318. https://doi.org/10.1016/S0010-8545(98)00260-4.
